# Collagen processing is essential for germ cell identity

**DOI:** 10.1242/bio.062198

**Published:** 2025-11-20

**Authors:** Sydney Roman, Nathalie Oulhen, Gerardo Reyes, Brenno Masina, Gary Wessel

**Affiliations:** ^1^ Department of Molecular and Cellular Biology, Brown University, 185 Meeting St., Providence, RI 02912, USA; ^2^ Laboratory of Organic Chemistry, D-CHAB, ETH Zurich, Vladimir-Prelog-Weg 3, 8093 Zurich, Switzerland

**Keywords:** Lysyl oxidase, Collagen, Germline inheritance, Glycolysis, Extracellular matrix, *Strongylocentrotus purpuratus*, Metabolism

## Abstract

The extracellular matrix (ECM) is a critical component of embryonic development, providing both structural support and a dynamic signaling environment for cell migration, adhesion, and tissue organization. Collagen, the most abundant protein in the ECM, is crosslinked by the enzyme lysyl oxidase (LOX), and that activity plays a pivotal role in creating support throughout the ECM. Dysregulated LOX activity disrupts the mechanical integrity of the ECM. Sea urchins offer a robust model for studying LOX function and ECM dynamics in embryonic development due to their rapid, transparent development and traceable cell lineages. Previous studies using the pan-monoamine oxidase/LOX inhibitor β-aminopropionitrile suggested an essential role of LOX activity in sea urchin gastrulation and maintenance of ECM integrity. Here, we integrate newly developed and traditional LOX inhibitors, with a translation blocking morpholino antisense oligonucleotide to a specific lysyl oxidase, and chemoselective fluorescent probes to LOX oxidation products, all to test the role of the ECM in development and germ cell formation. The primordial germ cells in this animal are believed to be committed at the fifth cell division as small micromeres by inheritance of yet unknown molecular constituency. We find that LOX activity is essential for an instructive environment in the development of a germ line, even though the fate of that germ line in the sea urchin is predetermined. Our findings provide insight into the dynamic interplay between ECM remodeling, gene expression, and metabolism, offering a more profound understanding of the role of the ECM in development and germ cell identity.

## Teaser

**Summary:** Germ cells fated by inheritance still read the collagenous extracellular matrix during fate determination and have plastic transcriptional responses.

## INTRODUCTION

Embryonic development is highlighted by orchestrated intercellular signaling, cell migration, and cell fate determinations. The extracellular matrix (ECM) is thought to play a vital role in these processes through its structural properties and intermolecular interactions (Kular et al., 2014). Proteins and glycan polymers that make up the ECM form a scaffold to create an environment for movement and signaling between cells. Among the diverse proteins comprising the ECM, collagen is the most abundant and serves as a key structural and functional component (Wu et al., 2025). While collagens have historically been considered for their biomechanical properties, it is now clear that they also function in highly selective and impactful intermolecular interactions By virtue of their various family members (>42 different genes in humans) and their ability to interact and form a complex meshwork, it is now appreciated that the collagen family is dynamic and influential on cellular physiology, fate, and positioning (Chan et al., 2008). One need only look at the many collagen-based human disorders that have been diagnosed to appreciate collagen functionality (Marfan's, Ehlers-Danlos, osteogenesis imperfecta) (Myllyharju and Kivirikko, 2001).

Collagen is also paradigmatic of post-translational processing, key to transitioning a soluble protein into an insoluble matrix. Procollagen is first secreted by cells into the ECM, where the N- and C-terminal ends are selectively cleaved to trigger their assembly into collagen fibrils. These fibrils are stabilized by covalent crosslinking reactions, e.g. a Schiffs-base reaction. The enzyme lysyl oxidase (LOX) catalyzes this crosslinking reaction between an amine group and the oxidized lysine residues on collagen fibrils. The LOX reaction results in an aldehyde bearing lysine residue, allysine, and releases hydrogen peroxide (H_2_O_2_) as a byproduct (Wu et al., 2025; Dolmatov and Nizhnichenko, 2023). Allysine further reacts with neighboring allysines to form covalent crosslinks, which enhance the mechanical stability and tensile strength of collagen fibrils. Dysregulated LOX activity compromises the ECM and its mechanical integrity, contributing to enhanced disease progression including metastasis, fibrosis, and cardiovascular disease (Kumari et al., 2017). This is the canonical LOX activity.

The presence of a specific catalytic domain distinguishes the family of LOX proteins from other enzymes, as this domain is crucial for their enzymatic function (Grau-Bove et al., 2015). All LOX proteins share this conserved catalytic domain near the C-terminus, which facilitates deaminacetylase activity (Zaffryar-Eilot and Hasson, 2022). This domain includes a copper-binding site, lysine tyrosylquinone, and a cytokine receptor-like domain. LOX is a copper-dependent enzyme, which acquires copper intracellularly. This copper binding motif is dependent on a conserved amino acid sequence within the catalytic domain of LOX proteins and is required for the activation of the lysyl tyrosyl quinone binding domain (LTQ) (Zaffryar-Eilot and Hasson, 2022). The LTQ is essential for LOX oxidation which results in the crosslinking of collagen fibrils (Guo et al., 2016). The last conserved portion of LOX's enzymatic domain is a cytokine-like receptor domain (CRL). The function of the CRL is not well understood, but it may facilitate ECM interactions (Guo et al., 2016). Together, the activation of these three domains induces the enzymatic activity of LOX by creating highly reactive aldehydes, which then spontaneously form crosslinks (Yang et al., 2020).

In contrast, the N-terminal regions of LOX proteins are highly variable, with LOX and LOX-like 1 (LOXL-1) domains differing significantly from LOX-like 2 (LOXL-2), LOX-like 3 (LOXL-3), and LOX-like 4 (LOXL-4) gene products. LOX and LOXL-1 are secreted in an inactive form that require cleavage by BMP-1 for activation, where it is either transported back into the nucleus (by yet unknown means) to regulate transcription or into the ECM to enzymatically crosslink collagen. LOXL-2, LOXL-3, and LOXL-4 are secreted as active proteins that contain repeat scavenger receptor cysteine-rich (SRCR) domains (Rodriguez-Pascual and Rosell-Garcia, 2018). The SRCR domain is ancient, highly conserved, and hypothesized to govern cell adhesion and cell signaling facilitated through protein-protein interactions (Sarrias et al., 2004; Wu et al., 2024; Zaffryar-Eilot and Hasson, 2022).

To further understand LOX function, researchers have extensively examined its enzymatic activity, often using β-aminopropionitrile (BAPN), a well-established irreversible LOX inhibitor. While it has been shown to successfully inhibit LOX by competitive inhibition, it may also inhibit enzymes with similar characteristics, including other monoamine oxidases (Hajdu et al., 2018). Newly developed molecules have been shown to inhibit LOX with greater specificity, such as PXS-4787 (Chitty et al., 2023). This pharmacological inhibitor selectively inhibits LOX activity while not affecting other oxidase types (Hiebert et al., 2024).

While most research focuses on the role of LOX in disease using mouse models, human biopsies, and cell cultures, sea urchins have been used to study similar processes during early development. Previous studies have used BAPN to inhibit LOX activity in developing sea urchin embryos (Benson et al., 1991; Wessel and McClay, 1987). These studies demonstrated that LOX is essential for gastrulation, as they observed a general failure in tissue morphogenesis, suggesting that collagen crosslinking is critical for proper development.

Sea urchins develop through a fixed developmental program using cell interactions and maternal deposits in the egg to guide various cell fate decisions (Davidson et al., 2002; McClay, 2011). This includes the primordial germ cells (PGCs), which eventually become the stem cells to form the egg and sperm in the adult (Inoue et al., 1992; Wessel et al., 2014a,b). These cells can readily be tracked through larval development, beginning at fertilization. In *Strongylocentrotus purpuratus* (the purple sea urchin or Sp, as we will refer to it later on), embryos divide to form a blastula in less than 24 h. By the second day, the gastrula has formed which is the invagination of the endoderm (the future gut of the larva) to eventually form the opening of the mouth, characteristic of this deuterostome. By 72 h post-fertilization, the embryo transitions into a triangular, swimming, and feeding larva.

Sea urchins rely on an inherited mechanism of germ cell specification, similar to the germ cell development in *Drosophila melanogaster*. The sea urchin though in contrast, does not have germ plasm, or concentrations of mitochondria, nor enriched regions of germ factor protein or RNA. This inherited reproductive mechanism is in contrast to how other echinoderm species function, such as the sea star, *Patiria miniata*, which uses inductive signaling to specify germ cells using similar mechanisms as in mammals for PGC formation. In *S. purpuratus*, the primordial germ cells are formed after two asymmetric cell divisions at the 4th and 5th cleavages (Campanale et al., 2014). The first asymmetric cleavage results in 4 macromeres and 4 micromeres. The second one divides the 4 micromeres into 4 large micromeres and 4 small micromeres; the latter are the PGCs (Oulhen et al., 2022). The PGCs are first quiescent with low cell cycle activity, transcriptional and translational activity, low RNA degradation activity, and low mitochondrial activity (Oulhen et al., 2017). They regain their activity following gastrulation as they translocate to the top of the invaginating archenteron (Oulhen et al., 2017). They then separate into the left and the right sides, which are tightly associated with the future coelomic pouches. The PGCs in the left coelomic pouch will become part of the adult rudiment, the structure that will form the juvenile sea urchin after metamorphosis, while those on the right side will undergo apoptosis (Oulhen et al., 2017).

Here, we utilized multiple methods to interrogate LOX function to understand the ECM's role in dynamically orchestrating cell development. By observing phenotypic, molecular, and metabolomic changes in LOX-inhibited embryos, we aimed to test whether the fundamental properties of the ECM in an embryo influence germ cell identity.

## RESULTS

### Multiple LOX genes are present in the sea urchin *S. purpuratus* genome

**Fig. 1.**
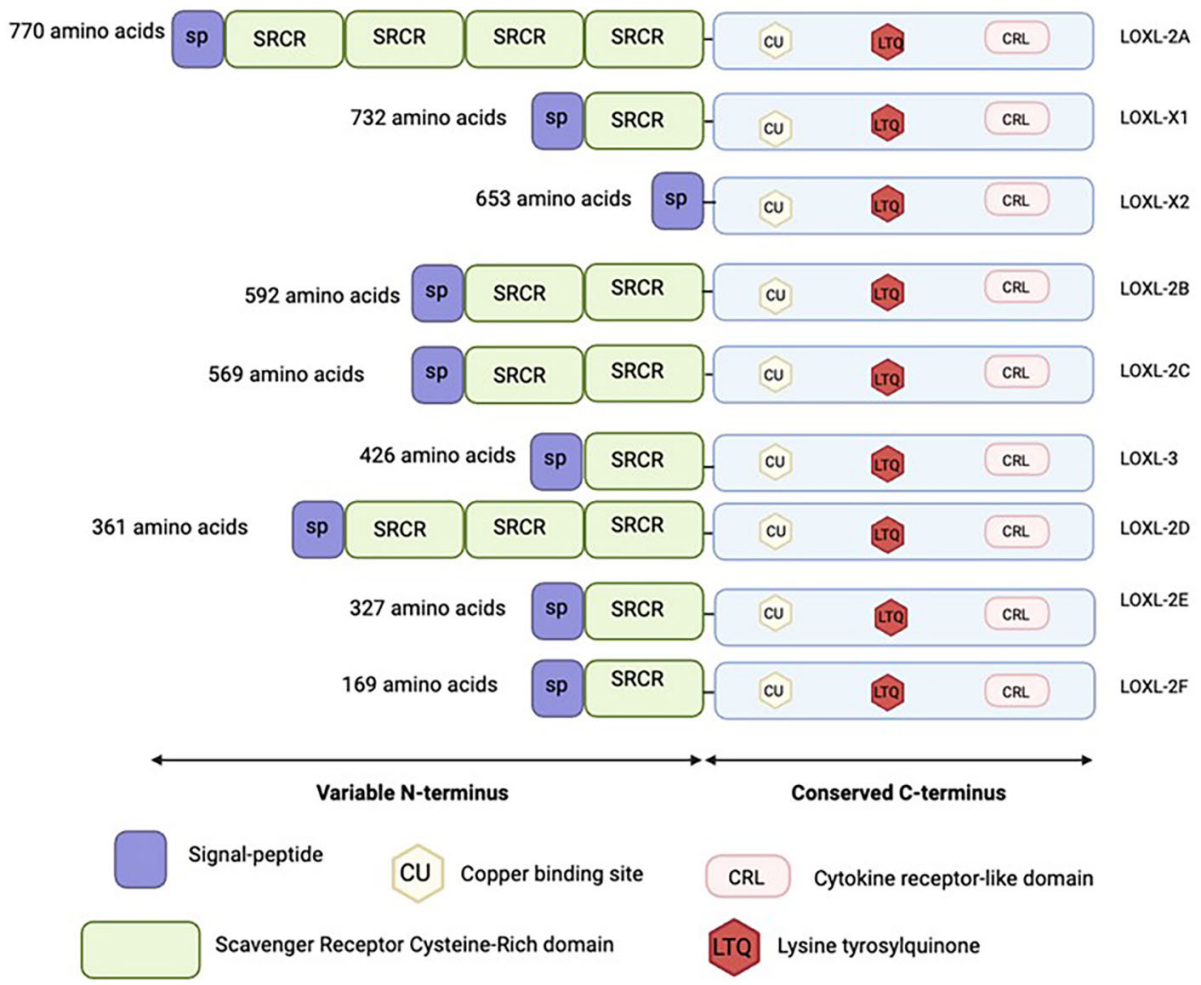
***S. purpuratus LOX* isoforms.** Eight LOX genes were identified on Echinobase and contain the characteristic LOX domain (Telmer et al., 2024). One of the genes encodes two different isoforms (LOXL-X1 and LOXL-X2). They all contain a conserved C-terminus domain including the copper binding site, lysine tyrosylquinone, and cytokine receptor-like domain. The N-terminus is variable with differing number of SRCR domain repeats. LOXL(X1/X2) was chosen for further research due to its expression in early development (Fig. 2A). Further gene identification is available in Fig. S1. Created in BioRender by Oulhen, N. (2025). https://BioRender.com/ywfnjqh. This figure was sublicensed under CC-BY 4.0 terms.

Eight different LOX genes were identified in the genome of *S. purpuratus* on Echinobase. These genes were blasted on NCBI to test their identity, and their protein domains were characterized (Fig. 1; Fig. S1). All of these genes encode LOX proteins that contain the highly conserved C-terminal domain essential for their enzymatic crosslinking activity (Grau-Bove et al., 2015). One of the LOX genes was found to encode two different protein isoforms: LOXL-X1 and LOXL-X2. LOXL-X1 is slightly longer than LOXL-X2 and, within those extra nucleotides, encodes the scavenger-receptor cystine rich (SRCR) protein domain, which is believed to have functions within protein-protein interactions (Fig. S2). This domain is characteristic of the LOXL2-4 proteins from all other species. While LOXL-X2 does not contain this SRCR domain, all the other LOX proteins identified did have this domain (Fig. 1). Mammalian LOX and LOXL1 (proteins with the propeptide sequence that is cleaved for enzymatic activation) have not been identified in sea urchin, as none of these Sp LOX genes contain this propeptide domain.

**Fig. 2.**
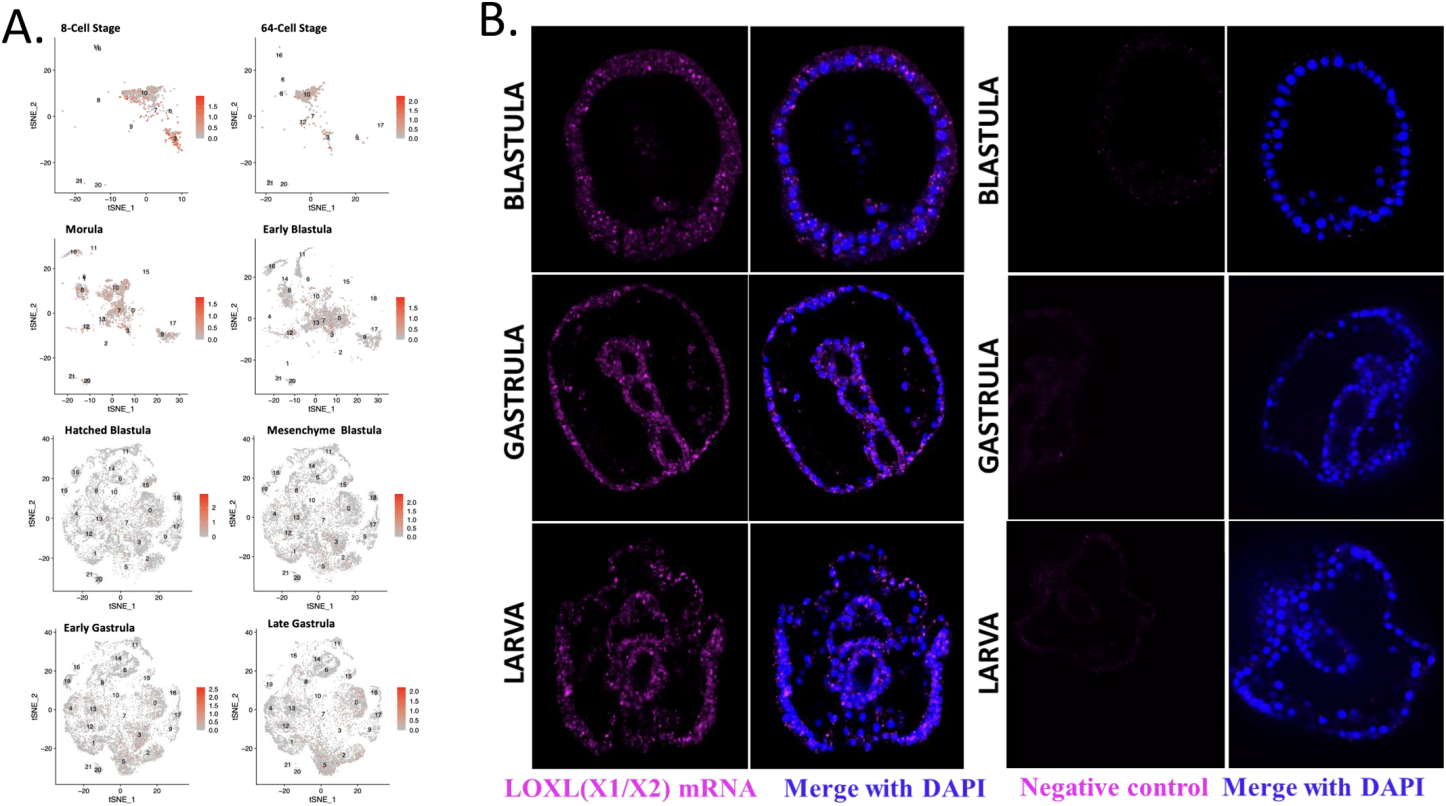
**LOXL(X1/X2) expression in early developmental stages.** (A) Single-cell RNA-sequencing analysis shows the expression of LOXL(X1/X2) throughout early sea urchin development, with red dots representing individual cells expressing LOXL(X1/X2) mRNA. The beginning of development shows strong expression. This expression decreases over development time. (B) LOXL(X1/X2) RNA *in situ* hybridization. Primers were designed against the LOXL(X1/X2) sequence representing the most strongly expressed LOX according to Echinobase and single-cell RNA-sequencing data (Telmer et al., 2024; Foster et al., 2021). The expression of LOXL(X1/X2) is not specific to one region of the embryo and is expressed throughout multiple developmental timepoints. LOXL(X1/X2) *in situ* RNA hybridization in pink, DNA in blue. Embryos are approximatively 100 μm in diameter.

### At least two LOX genes are expressed in the early development of the sea urchin

The expression of the LOX mRNAs was tested using our previously published single-cell RNA-sequencing (RNA-seq) dataset (Foster et al., 2021). LOXL(X1/X2) had the highest RNA expression level compared to the other LOX genes during early development. It is expressed abundantly throughout early development in the single-cell RNA-seq dataset, appearing in almost all the different clusters of cell types and states (Fig. 2A). As developmental time continues, LOXL(X1/X2) expression decreased until the larval stage (Fig. 2A). We cannot distinguish the difference between LOXL-X1 and LOXL-X2 in the single-cell RNA-seq dataset, as they come from the same gene. The Nanostring dataset from Echinobase does differentiate these differentially spliced isoforms and shows both LOXL-X1 and LOXL-X2 are expressed in early development, but LOXL-X1 is expressed at a much higher level (Fig. S3) (Telmer et al., 2024).

Of the other LOX genes identified, only LOXL-2B was expressed in the early time points of development. LOXL-2B is expressed ubiquitously before gastrulation, then its expression decreases (Fig. S4). As observed from the Nanostring data, the transcript levels (TPM) of LOXL-2B are expressed at half the value of LOXL(X1/X2) (Telmer et al., 2024). The other six LOX genes were not detected in these datasets and will not be further analyzed in this study.

A probe recognizing both LOXL-X1 and LOXL-X2 was used to test their expression in embryonic development. By RNA *in situ* hybridization, LOXL(X1/X2) mRNA is detected at a low level in blastula, gastrula, and larval stages, and it does not appear to be enriched in any specific cells (Fig. 2B). Currently, we do not have any functional antibodies to recognize the LOX family members to compare this work with protein expression. Altogether, these results suggest that two LOX genes are expressed during early sea urchin development and could play a role in regulating the extracellular matrix.

### BAPN and PXS reduce LOX activity and collagen crosslinking in sea urchin embryos

**Fig. 3.**
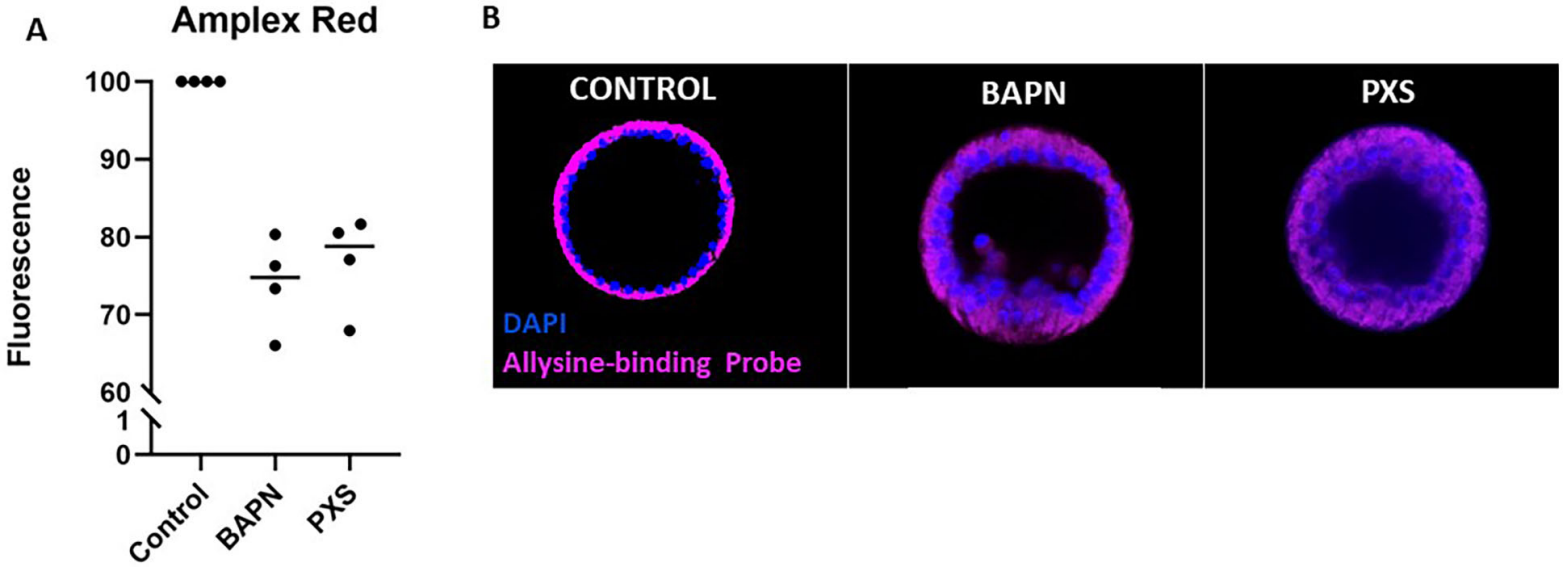
**BAPN and PXS reduced LOX activity and collagen crosslinking in sea urchin embryos.** (A) The Amplex Red assay quantifies the fluorescence created by hydrogen peroxide, which is a byproduct of the LOX reaction. Amplex Red reagent was added to gastrula-stage embryos, and a microplate reader analyzed fluorescence after 30 min. There is a 25% and 20% decrease between the LOX-inhibited samples, BAPN and PXS at 500 µM, respectively, and the controls. This shows that in LOX-inhibited samples, the hydrogen peroxide released is reduced. (B) Allysine-binding chemical probe (pink; DNA in blue) illuminates areas of active collagen deposition and cross-linking. Control embryos display a strong and dense fluorescent signal from the chemical probe around the apical layer of the embryo. Reduced fluorescence is observed in LOX-inhibited embryos indicating reduced collagen cross-linking. Embryos are approximatively 100 μm in diameter.

To test the function of LOX in sea urchin development, two small-molecule inhibitors, BAPN and PXS-4787, were used (Butler et al., 1987; Chaudhari et al., 2023). A functional assay was first used to test the effect of these inhibitors on LOX activity. Amplex Red is a fluorogenic substrate that is oxidized into the fluorophore resorufin in the presence of H_2_O_2_ (Fernando Rodriguez-Pascual, 2022). H_2_O_2_ is naturally created by various cellular processes, but is particularly prominent as a byproduct from the LOX reaction (Fernando Rodriguez-Pascual, 2022). A lower fluorescence value was expected in LOX-inhibited samples, as inhibited LOX activity would result in less collagen crosslinking and less H_2_O_2_ production. Embryos at different stages were treated with BAPN or PXS-4787 and incubated for 24 h before performing the Amplex Red assay. The fluorescence was measured at multiple time points after adding the Amplex Red substrate, but for all developmental stages, the 30-min time point showed the greatest change in fluorescence (not shown). Blastula, gastrula, and larval stages all showed the same trend of decreased, but not complete cessation of H_2_O_2_ production. In gastrula, for example, we saw an approximate decrease in fluorescence of 25% and 20% in BAPN- and PXS-treated samples, respectively (Fig. 3A). As BAPN is a more general monoamine oxidase inhibitor, it is possible that other monoamine oxidase enzymes are also targeted by the inhibitors, resulting in a greater decrease in fluorescence (Fig. 3A). Since PXS is solely inhibiting LOX activity, it leads to a smaller decrease in fluorescence, although still significant compared to control values (Fig. 3A) (Hiebert et al., 2024). By comparing and normalizing the treated values to the control, the non-LOX produced peroxide was accounted for. When adding BAPN to embryos and running the Amplex Red experiment immediately, there was no change in the levels of fluorescence (not shown). This occurred because BAPN works by a time-dependent inhibition to block the LOX activity (Chaudhari et al., 2023).

Given that the Amplex Red assay measures H_2_O_2_ generated during LOX-mediated cross-linking, the assay is prone to interference from other endogenous sources of H_2_O_2_. To assess LOX-mediated cross-linking more directly, we employed a chemical Bioprobe that binds selectively to allysine residues generated by LOX on collagen (Hiebert et al., 2024; Aronoff et al., 2021). This will only mark the processing of immature collagen, not mature collagen. The probe is equipped with a standing fluorophore, allowing for direct visualization of active collagen deposition and cross-linking areas. The signal seen from the allysine-binding probe is concentrated around the apical region of the embryo's epithelial cells (Fig. 3B). This signal decreased in BAPN and PXS-treated embryos, showing a decrease in collagen processing in LOX-inhibited samples (Fig. 3B).

The Amplex Red assay provided quantitative measurement of reduced enzymatic activity upon inhibitor treatment, while the Bioprobe allowed for visualization of LOX inhibition and collagen crosslinking reduction in live embryos. Together, these complementary approaches validate the efficacy of the inhibitors, reinforcing their potential as tools for studying LOX function during sea urchin development.

### Inhibition of LOX activity affects embryonic development in the sea urchin

To test if LOX activity is necessary for proper embryogenesis and overall embryonic integrity, embryos were incubated with BAPN and PXS at a low dose [100 µM] or a high dose [500 µM]. Embryos were treated with the inhibitor by time windows of 24 h: fertilization to blastula, or blastula to gastrula, or gastrula to larva (Fig. S5).

**Fig. 4.**
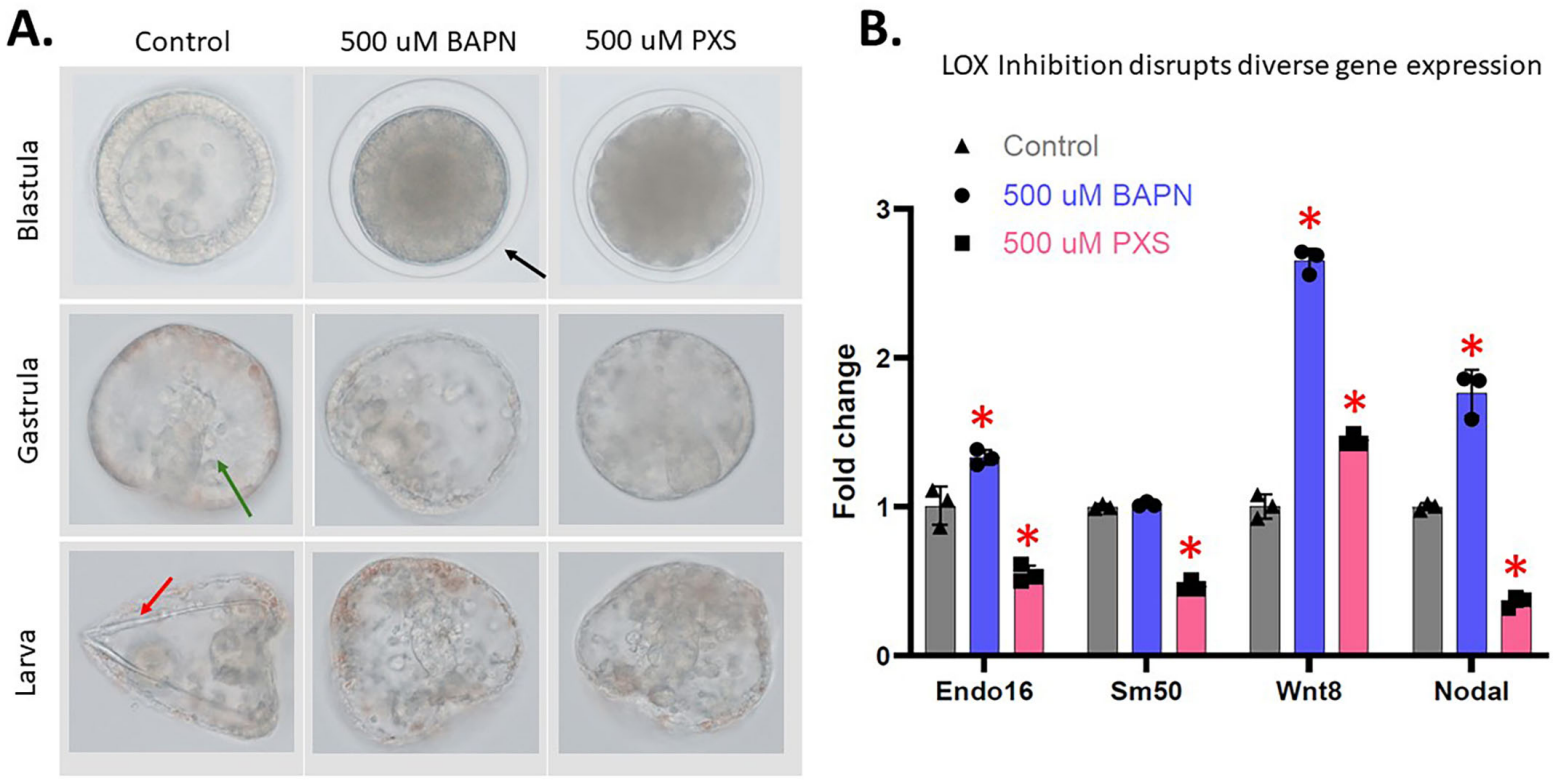
**Inhibition of LOX activity affects embryonic development in the sea urchin.** (A) Phenotypic observation of LOX-inhibited embryos through BAPN and PXS. 500 µM BAPN and 500 µM PXS showed a similar phenotypic effect, resulting in a developmental delay in the blastula stage marked by the fertilization envelope, shown with a black arrow. In gastrulation, the gut invaginates (green arrow) in control embryos, while LOX inhibited embryos show a lack of the gut formation. The control larval stages form a prism shape with skeletal rods (shown with a red arrow), and LOX-inhibited embryos lack a triangular phenotype and skeletal rod formation. B) RNA accumulation by qPCR of cell type markers important in development. These embryos were dosed at blastula stage and allowed to absorb the inhibitor for 24 h before being collected and analyzed at gastrula stage. BAPN and PXS were used at 500 µM. *t*-tests were used for statistical analysis (*P<0.05). The *y*-axis of the graph represents the fold change in RNA expression in the LOX-inhibited embryos compared to a control normalized to 1 at the same time point in development. Embryos are approximatively 100 μm in diameter.

When embryos were treated with 500 µM of BAPN or PXS at fertilization and analyzed 24 h later at the blastula stage, most of the embryos had been developmentally delayed (Fig. 4A). At the gastrula stage, after being dosed 24 h prior, the forming archenteron appeared flaccid, or barely visible (Fig. 4A). When embryos were dosed at the gastrula stage and imaged 24 h later at the larval stage, many embryos still had a spherical phenotype and had not formed the distinctive prism shape of the larval stage (Fig. 4A).

To better characterize the role of LOX activity in embryonic development, the mRNA expression of various cell type markers of the sea urchin were tested by quantitative PCR (qPCR) in gastrula-stage embryos (dosed at blastula stage): an endoderm marker, Endo16 (Nocente-McGrath et al., 1989); an ectoderm marker, Nodal (Molina et al., 2013) involved in left-right asymmetry; Sm50, a skeleton lineage marker (Benson et al., 1987, 1986); and Wnt8, an endomesoderm signaling molecule (Stamateris et al., 2010) (Fig. 4). The qPCRs at the gastrula stage show that the expression of Endo16, Wnt8 and Nodal is affected by LOX inhibition via BAPN compared to control embryos (Fig. 4B). Most transcripts are elevated when exposed to BAPN, revealing perhaps a compensatory effect, but high doses of PXS show decreased transcript levels in multiple different germ layers (Fig. 4B). The transcriptional differences between BAPN and PXS may result from the different levels of specificity between the two inhibitors.

**Fig. 5.**
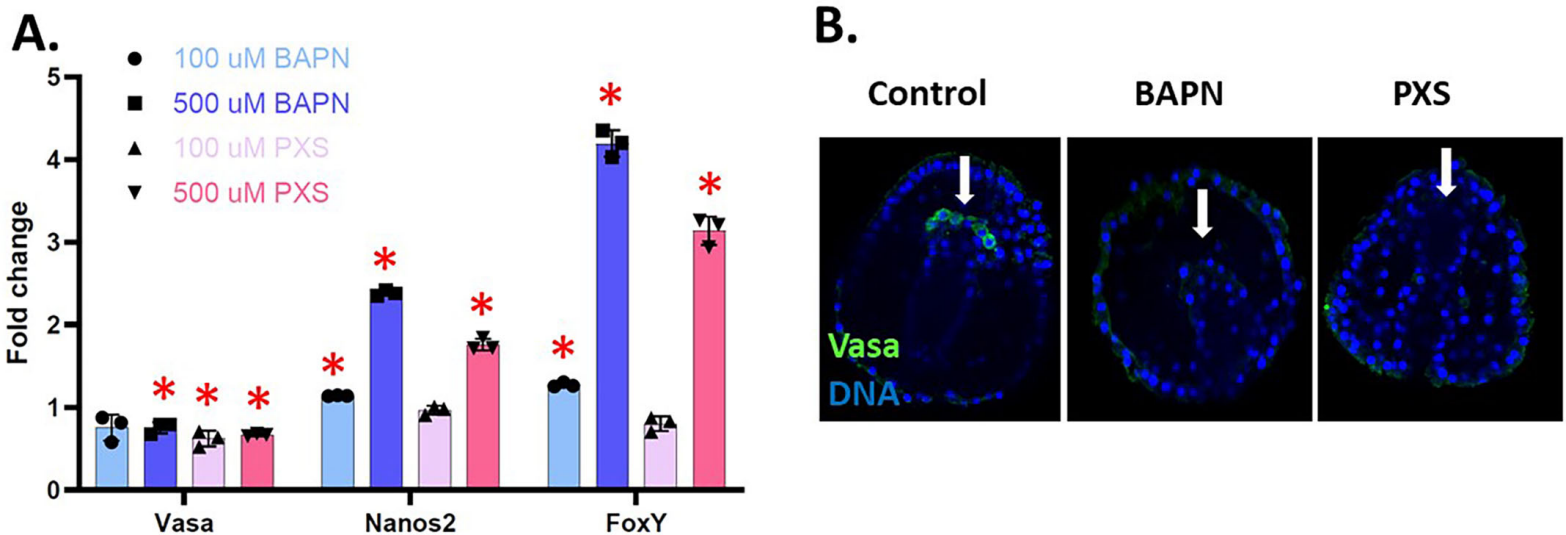
**Effect of LOX inhibition on germline-related genes in gastrulae.** (A) RNA accumulation by qPCR data shows a significant decrease in Vasa expression in embryos treated with 100 µM PXS and BAPN/PXS at 500 µM compared to controls. Nanos2 is upregulated at 500 µM of both BAPN and PXS. FoxY expression significantly increased at high doses of BAPN/PXS by a fold change of over 3. *P<0.05. The *y*-axis of the graph represents the fold change in RNA expression in the LOX-inhibited embryos compared to a control normalized to 1 at the same time point in development. (B) Vasa protein (green) is undetectable after 500 µM of BAPN or PXS treatment (arrows). DNA is labeled in blue. Embryos are approximatively 100 μm in diameter.

Vasa and Nanos2 are germline markers essential for germline specification and maintenance. Vasa is an RNA helicase (Voronina et al., 2008). Nanos2 is a translational regulator essential for PGC quiescence in this animal (Oulhen and Wessel, 2016). FoxY regulates Nanos2 transcription in the Veg2 mesoderm, the cells surrounding the PGCs (Song and Wessel, 2012). By qPCR, compared to the controls, Vasa RNA expression decreased in the LOX-inhibited embryos while the expression of FoxY and Nanos2 RNAs increased significantly when drugs were used at 500 µM (Fig. 5A). *In situ* RNA hybridization of Vasa, Nanos2*,* and FoxY in BAPN-treated gastrula support these qPCRs (Fig. S6). These *in situ* hybridizations show that the PGCs translocated to the normal location on top of the archenteron but experienced changes in gene expression that could affect their identity as germ cells.

The reduction in Vasa RNA expression observed in BAPN or PXS-treated embryos is also associated with a decrease in its protein expression. (Fig. 5B). Based on hybridization results (Fig. S6), we conclude that the PGCs are still present, but lack Vasa protein. Recalling that Vasa is regulated dramatically by post-translational mechanisms (Gustafson et al., 2011), we have yet to pinpoint which step prevents Vasa detection by LOX inhibition.

**Fig. 6.**
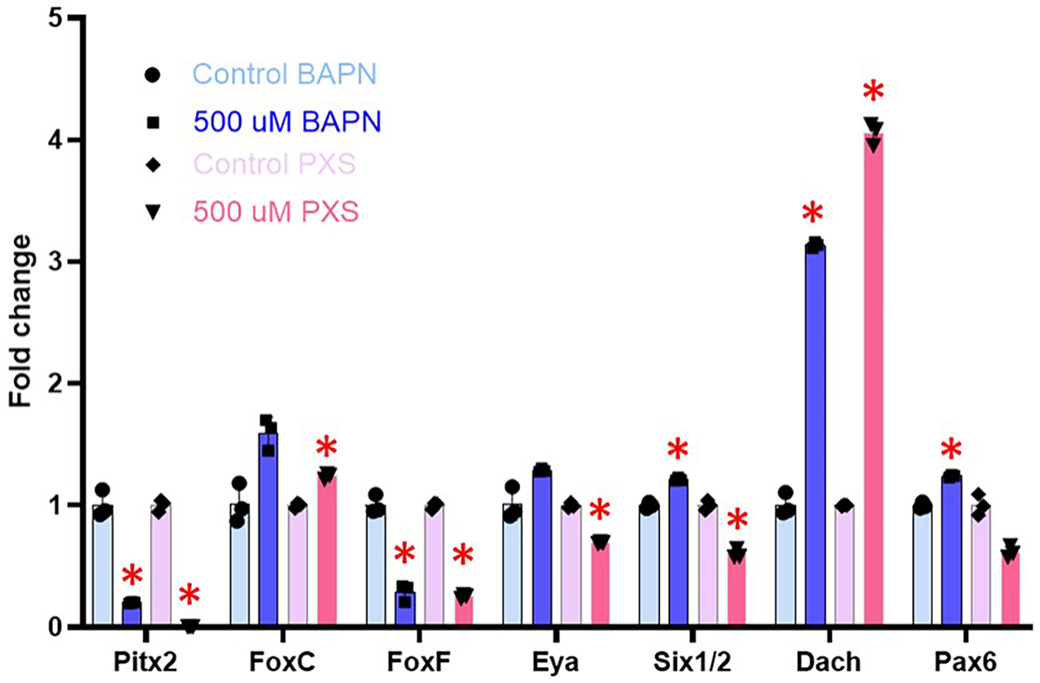
**Effect of BAPN and PXS on coelomic pouch genes.** RNA accumulation of coelomic pouch genes analyzed by qPCR at gastrula stage. Embryos were treated with drugs from blastula to gastrula stage. *P<0.001. The *y*-axis of the graph represents the fold change in RNA expression in the LOX-inhibited embryos compared to a control normalized to 1 at the same time point in development. BAPN and PXS were used at 500 µM.

At the end of gastrulation, the PGCs reside within the left and right coelomic pouches. The PGCs on the left pouch will participate in the formation of the rudiment. Genes encoding the transcription factors Eya, Six1/2, Dach, and Pax6 are known to be expressed in the left coelomic pouch, while Pitx2 is expressed in the right coelomic pouch (Duboc et al., 2005). Compared to the controls, the expressions of Pitx2 and FoxF are significantly downregulated in embryos treated with BAPN or PXS, while the expression of Dach is significantly upregulated (Fig. 6). These findings highlight the dynamic transcriptional regulation within each coelomic pouch and their diverse response to the BAPN and PXS treatments.

### Specific knockdown of LOXL(X1/X2) affects broad cell types

Out of the multiple LOX genes present in the sea urchin database, LOXL(X1/X2) has the highest mRNA expression in the early stages of development and is well expressed in the single-cell data (Fig. 2). This provided a good target for a translation blocking morpholino (MO). The 5′UTR sequence needed validating, so we sequenced three amplicons around the initiating ATG. Following sequence validation (Fig. S8), a MO was designed.

**Fig. 7.**
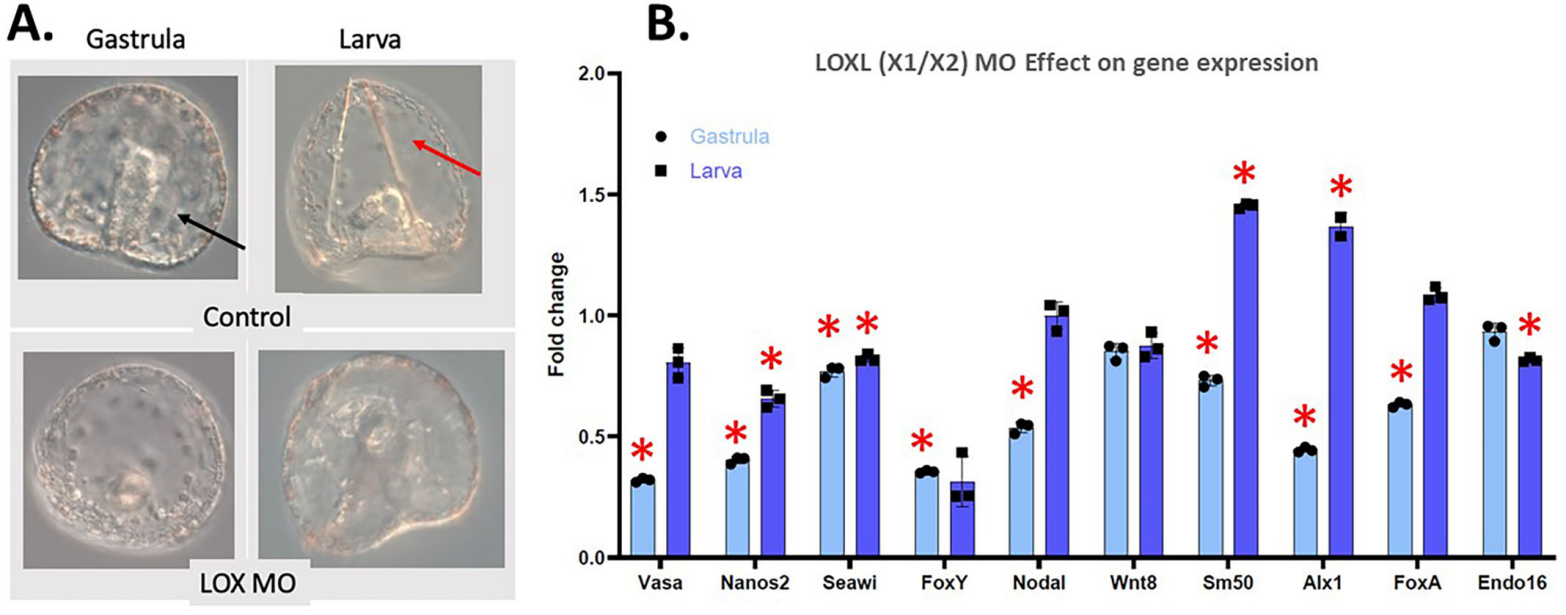
**Knockdown of LOXL(X1/X2) affects all germ layers at gastrula and larval stage.** (A) The gastrula stage LOXL(X1/X2) MO embryos are unable to invaginate to create a gut (black arrow in controls) and have a developmental delay in the larval stage. LOXL(X1/X2) MO larval stage are also lacking skeletal rods (red arrow in control). (B) RNA accumulation by qPCR of important cell type markers in gastrula and larval stage LOXL(X1/X2) MO embryos show significant changes in most genes. The *y*-axis of the graph represents the fold change in RNA expression in LOXL(X1/X2) MO embryos compared to a control normalized to 1 at the same time point in development. Dark-blue bars represent gastrula stage embryos and light-blue bars represent the larval stage. *P<0.005. Embryos are approximatively 100 μm in diameter.

As LOXL-X1 and LOXL-X2 are isoforms from the same gene, the MO is predicted to block the translation of both isoforms. Knockdown of this LOXL(X1/X2) translation using a specific MO led to similar phenotypic effects as seen with the chemical inhibitions, but to a lesser extent (Fig. 7A). In early stages, there appears to be a developmental delay (not shown). In gastrula stage embryos, the gut is less robust and does not form correctly in LOXL(X1/X2) MO embryos (black arrow in Fig. 7A), supported also by the significant decrease in the expression of the endoderm marker FoxA (Fig. 7B). In larvae, skeleton defects were seen and completely lacking in many larvae (red arrow in Fig. 7A; same structure not seen in MASO larvae). This is also supported in the qPCR data, where skeletogenesis is altered, shown by the upregulation of Sm50 and Alx1 in the larval stage (Fig. 7B). We interpret this upregulation in transcript accumulation for LOXL(X1/X2) as a compensatory activity for the larva not making a skeleton. LOX inhibition also had an effect on the germ cell-related genes. At the gastrula stage, Vasa, Nanos2, Seawi, and FoxY are all significantly downregulated in comparison to controls (Fig. 7B). The larval stage showed a downregulation of Nanos2 and Seawi RNAs (Fig. 7B).

### LOX inhibition affects other ECM proteins

**Fig. 8.**
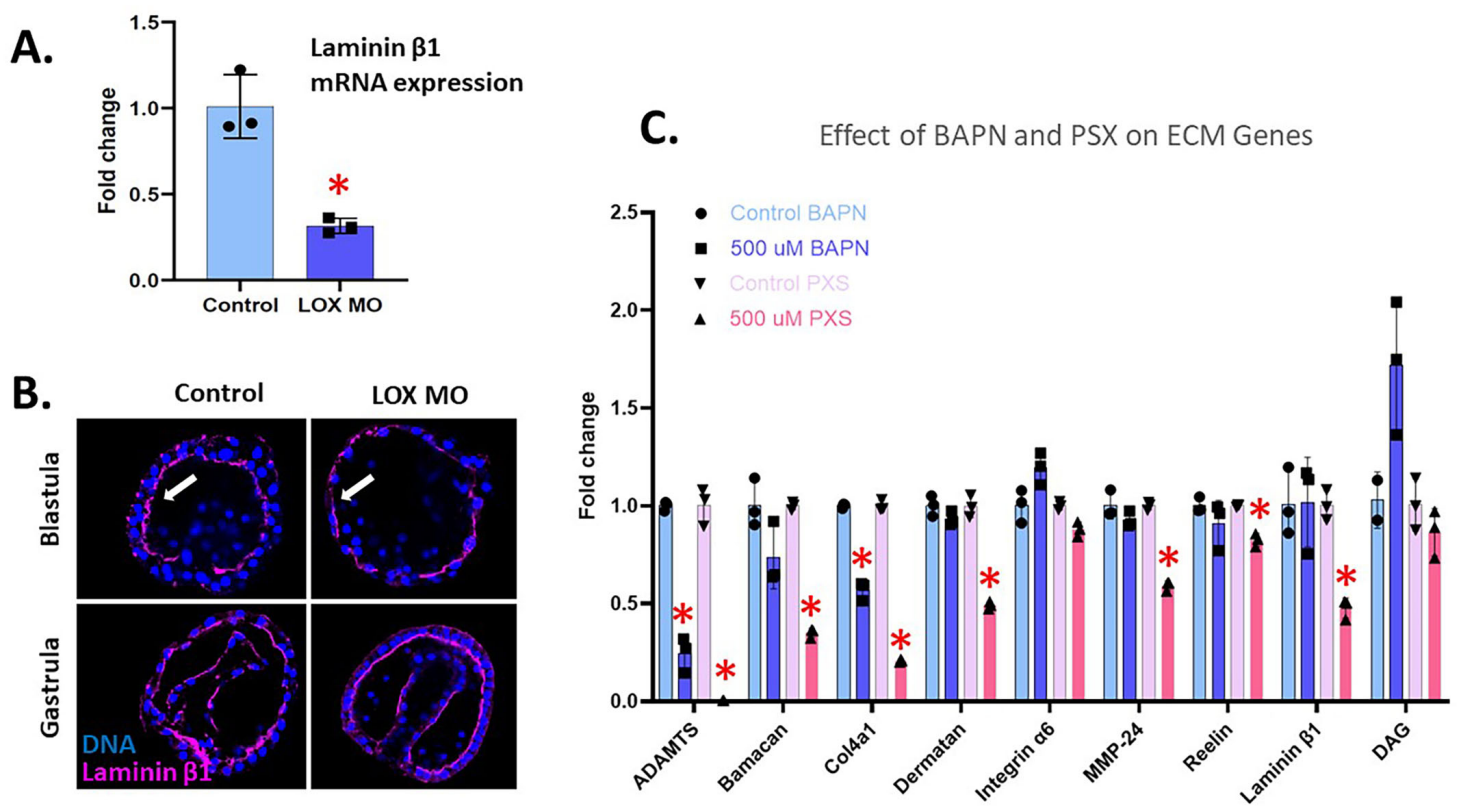
**Effect of LOX inhibition on other ECM genes.** (A) Laminin-beta-1 expression in LOXL(X1/X2) MO gastrula-stage embryos through qPCR analysis. The expression of laminin-beta-1 significantly (*P*<0.05) decreased in embryos injected with LOXL(X1/X2) MO compared to the control. (B) Immunofluorescence of laminin-beta-1 (pink) and DNA (blue) show a slight decrease in expression in the LOXL(X1/X2) MO embryos complementary to the qPCR data. (C) RNA accumulation by qPCR analysis of ECM-associated genes in 500 µM BAPN- or PXS-treated embryos at gastrula stage. Embryos were treated with drugs from blastula to gastrula stage. ADAMTS and Collagen4a1 are significantly downregulated in both BAPN- and PXS-treated embryos. *P<0.001. The *y*-axis of the graph represents the fold change in RNA expression in the LOX-inhibited embryos compared to a control normalized to 1 at the same time point in development. Embryos are approximatively 100 μm in diameter.

Laminin is an ECM protein that connects the cell to the ECM. It does this by binding between collagen and integrin membrane proteins (Aumailley, 2013). In LOXL(X1/X2) MO embryos and in PXS-treated embryos, we see a significant decrease in the Laminin beta1 mRNA expression compared to control embryos at gastrula stage (Fig. 8A,C). Using an antibody against Laminin beta1, control blastula show the presence of this protein on both the basal and the apical lamina but this organization is lost in the LOXL(X1/X2) MO embryos (Fig. 8B). However, laminin beta1 RNA (Fig. 8C) and protein expression (Fig. S7) are not affected by the BAPN treatment. The LOXL(X1/X2) MO is specifically blocking the expression of one LOX gene, while BAPN blocks the activity of the proteins coded by all LOX genes, which may explain these differences. Many other ECM-related genes are affected by the loss of LOX activity through either BAPN or PXS treatment (Fig. 8C). Collagen IV (an essential collagen in basal lamina formation) and ADAMTS (a disintegrin and metalloproteinase with thrombospondin motifs) are significantly downregulated in both BAPN- and PXS-treated embryos (Boudko et al., 2018; Kelwick et al., 2015). Inhibiting one aspect of ECM modification, such as LOX activity, leads to widespread transcriptional changes, with multiple ECM-related genes being downregulated, especially after PXS treatment. This highlights the importance of transcriptional regulation in maintaining ECM integrity, as cells may activate compensatory mechanisms in response to structural disruptions. To investigate these effects, future work will include electron microscopy analysis of LOX-inhibited embryos to visualize how the ECM structure is altered.

### Effect of LOX inhibition on metabolism

The dramatic shifts in transcript accumulation seen with LOX inhibition suggested the embryos were impacted by more than RNA levels. We decided to test changes in metabolic profiles that may reveal compensatory mechanisms or disruptions in key biosynthetic pathways necessary for maintaining ECM homeostasis. Therefore, metabolomics analysis was performed on control, BAPN-treated, and PXS-treated embryos. LOX activity is mostly present in the ECM; however, other monoamine oxidases exist within the cell, generating distinct metabolites that can further inform our understanding of oxidative processes in embryonic development. By using an untargeted approach, all the metabolites produced could be analyzed without predefined or biased targets.

**Fig. 9.**
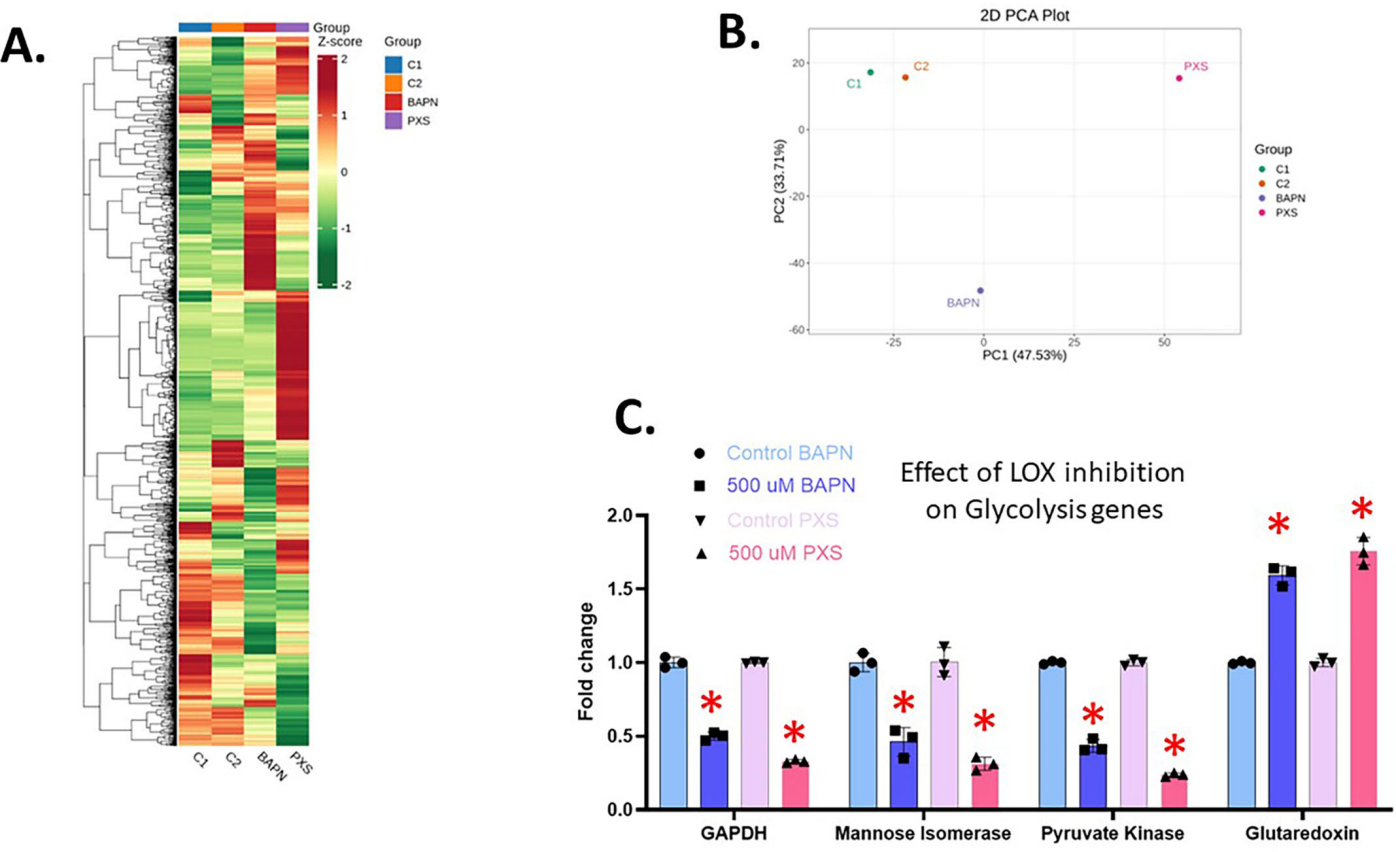
**Effect of LOX inhibition on metabolism.** (A) A heatmap showing differential metabolite profiles comparing two control samples (C1 and C2) with 500 µM BAPN- and PXS-treated samples. C1 and C2 are technical replicates from the same sample of embryos. Red bars indicate a positive Z score, showing upregulation of the specific metabolite, while green indicates downregulation. The tree shows the clustering of metabolites within similar pathways. (B) The PCA plot shows the similarity of metabolite composition between the four samples. C1 and C2 are clustered near each other, representing their similarity, while PXS and BAPN at 500 µM are separated from controls and each other. (C) RNA accumulation by qPCR of genes involved in glycolysis (a pathway influenced by LOX inhibition) at gastrula stage. Embryos were treated with drugs from blastula to gastrula stage. The RNAs coding for three enzymes involved in the pathway (GAPDH, Mannose Isomerase, and Pyruvate Kinase) are all downregulated with BAPN and PXS treatments. Glutaredoxin RNA, which codes for a protein that plays a role in glycolysis metabolism and regulation, is upregulated when LOX is inhibited. *P<0.005. The *y*-axis of the graph represents the fold change in RNA expression in the LOX-inhibited embryos compared to a control normalized to 1 at the same time point in development.

Overall, the heatmap and the principal complement analysis (PCA) plots show significant differences in the metabolites detected in controls and LOX-inhibited samples (Fig. 9A,B). Differences are also seen between BAPN and PXS, representing how a difference in their specificity can affect the metabolites produced. We point out that we were unable to perform additional replicates in this analysis for statistical significance, so we tested suggestive gene batteries from the metabolome. The main pathway enriched when we analyzed Kyoto Encyclopedia of Genes and Genomes (KEGG)-based differentially expressed metabolites is glycolysis (data not shown). We therefore tested by qPCR the expression of key genes involved in the glycolytic pathway to test if they could be linked with the changes seen in the glycolytic metabolites.

Pyruvate Kinase, Glutaredoxin, GAPDH, and Mannose Isomerase each stood out in our metabolic analysis, and we tested them by qPCR (Fig. 9C). Pyruvate Kinase generates pyruvate and ATP and has shown previously to be downregulated in the primordial germ cells in sea urchins (Furze et al., 2024). GAPDH catalyzes the only reductive step in glycolysis (Henry et al., 2015). Mannose Isomerase breaks down mannose to enter the glycolytic cycle (de Vasconcellos Racorti et al., 2024) and Glutaredoxin is a regulator of glycolysis (Lopez-Grueso et al., 2019). BAPN and PXS led to similar changes in RNA expression: enzymes of glycolysis are downregulated (Pyruvate Kinase, GAPDH, and Mannose Isomerase), while the glycolysis regulator is upregulated (Glutaredoxin) (Fig. 9C). These findings suggest that LOX inhibition disrupts glycolysis by downregulating key enzymes involved in energy production while upregulating a regulatory factor, altering the metabolism in the embryos. We do not currently know if these metabolic changes are cause/effect, correlative, or causative to the phenotypes in development and gene expression. Still, we now have a road map to better understand the role of LOX in metabolism.

## DISCUSSION

We have identified eight different LOX genes in the sea urchin genome and functionally tested the candidate with the greatest expression in embryos. They all contained the highly conserved C-terminal and characteristic domain required for their lysyl oxidase enzymatic activity (Cu binding domain, CRL, and LTQ) (Molnar et al., 2003). However, these *S. purpuratus* LOXs contain SRCR domain repeats, which is characteristic of mammalian LOXL2-4. These SRCR domains are less conserved and do not align properly between the LOX proteins. In mammals, only five LOX proteins are present (LOX, LOXL1-4). Many of them are tissue specific and play distinct roles within collagen remodeling. LOX and LOXL-1 mammalian proteins with the distinctive propeptide sequence have yet to be found in the *S. purpuratus* genome. This suggests that *S. purpuratus* may have evolved similarly complex LOX variants. Further investigation is needed to determine the evolution and the functional diversity of these LOX proteins and their specific roles in Echinoderm development and physiology.

Mapping sea urchin LOX genes using sequences from other species has proven challenging due to the extensive variation in LOX forms, including frequent duplications and rearrangements of protein domain structures. This makes it difficult to identify and compare LOX genes between species accurately. It is possible that additional LOX genes exist in the sea urchin genome. We found that the LOXL-X1/X2 gene encodes at least two different transcripts (X1 and X2), suggesting that additional LOX transcripts and/or proteins could also be synthesized from the other genes. LOXL-X2 mRNA is more expressed than LOXL-X1 in early development. This loss of the SRCR domain within the LOXL-X2 isoform could lead to a less stable protein, or perhaps a loss of function that is compensated by LOXL-X1. Further studies will include designing LOXL-X1 and LOXL-X2 specific antibodies to distinguish their individual protein expressions throughout embryogenesis.

In *S. purpuratus*, many of the LOX genes have very low RNA expression in the single-cell RNA-seq dataset of early development. Except for LOXL(X1/X2) and LOXL-2B, none of the other *S. purpuratus* LOX genes have detectable expression during early development; however, they could still be expressed at undetectable levels with the techniques utilized here. The expression of these other *S. purpuratus* LOX genes could be maintained at low concentrations upregulated on demand. Some of these LOX genes could instead be expressed in later stages in development. Future studies will include identifying the function of LOX in an adult sea urchin to better understand the homeostatic role of collagen crosslinking in the ECM. By using data created from careful dissection and RNA sequencing of each organ/structure from a *Lytechinus variegatus* sea urchin, we see which tissues of the adult urchin express LOX RNA (Pieplow et al., 2025). Although this is a different species of sea urchin, there is an orthologous gene for LOXL-2A, which is expressed in many adult tissues from the radial nerve (highest expression) to the ovary (lowest expression) (Fig. S9). These specific expression patterns suggest that LOXL-2A may play a role in maintaining the structural integrity of these adult tissues, highlighting the importance of LOX gene regulation in cellular homeostasis throughout the life of the sea urchin.

LOX can be regulated transcriptionally and post-translationally, as well as intracellularly and extracellularly. Intracellular LOX is most commonly regulated by hypoxia inducing factors (HIFs). These factors upregulate LOX transcription in the nucleus (Wei et al., 2020). It has been shown separately that both LOX and HIF can promote the growth of newly formed tissues. There may be a regulation between these two proteins, since mutations in HIFs can increase LOX expression, creating uncontrolled growth in breast cancer. When inactive LOX proteins are secreted into the ECM, they can be regulated by transforming growth factor (TGF-B), tolloid protein 1 (TLD-1), and fibronectin, which all promote its transcriptional activity and/or its activation (Wei et al., 2020). Stimulation from other factors, such as homocysteine and prostaglandin E2, have the opposite effect on LOX expression (Wei et al., 2020). Copper binding and BMP are also involved in LOX activity and play a role in the activation and regulation of LOX.

When LOX is inhibited, important germline markers such as Vasa, FoxY, and Nanos2 are all affected. Vasa is a DEAD-box RNA helicase enriched in the primordial germ cells (Voronina et al., 2008). In LOX-inhibited gastrula (by both BAPN and PXS), Vasa mRNA expression is downregulated, suggesting that the PGCs could be losing their identity as germ cells. FoxY is a transcription factor that plays a role in the formation of the coelomic pouch during normal development, a structure that contains the PGCs and needs a stable ECM to form properly (Song and Wessel, 2012). Nanos2 is a translational regulator required for the maintenance of primordial germ cells (Juliano et al., 2010). When the micromeres (precursors of the PGCs) are removed from sea urchin embryos, these embryos still develop into adults that have gonads with gametes (Yajima and Wessel, 2011). The hypothesis is that the Veg2 mesoderm (the somatic cells surrounding the PGCs at the gastrula stage) could create a new germline in these micromere-deleted embryos. FoxY has been shown to regulate Nanos2 expression in the Veg2 mesoderm in normal development. In LOX-inhibited embryos (100 µM BAPN, 500 µM BAPN, and 500 µM PXS), FoxY and Nanos2 expression is significantly increased by 3-fold and 2-fold, respectively. With inhibition of germ cell specification through the loss of Vasa, FoxY may be compensating and increasing the transcription of Nanos2 in the Veg2 mesoderm. Further studies will look at the specific location of Vasa, FoxY, and Nanos2 transcripts using fluorescent *in situ* RNA hybridization after BAPN and PXS treatments. Antibodies for these genes could also reveal their protein localization; however, protein changes could be more or less pronounced than the mRNA expression. In contrast to these results, FoxY and Nanos2 RNAs were downregulated when PXS was used at a low dose (100 µM), suggesting that the FoxY and Nanos2 compensation mechanisms might only be activated at a high dose of PXS. Sea urchins use an inherited mechanism to specify their germline. Here, we show that, despite the PGCs being set aside early in the development, the ECM still plays a major role for the maintenance of their identity. In contrast, sea stars use inductive mechanisms to specify their germline, and they rely on cell signaling at a later stage of development. Recent data have shown that sea star embryos incubated with BAPN also show defects in their germline gene expression (Reyes et al., 2025). Altogether, these data demonstrate how important the extracellular matrix is to maintain the identity of the primordial germ cells, independently of the mechanisms used for their specification.

LOX inhibition also affects the expression of other ECM genes. Laminin is an ECM glycoprotein that is important in the basal lamina and is essential for cell movement. It binds to cell surface receptors such as integrins and dystroglycan, mediating cell movement and migration (Aumailley, 2013). When LOX is inhibited in sea urchin embryos via a translation blocking MO or after PXS treatment, we see a significant decrease in laminin-beta-1 mRNA expression. However, it does not change in BAPN-treated embryos. This response to ECM instability raises the question of how different methods of LOX inhibition influence gene expression and developmental outcomes.

In addition, we observed that the LOXL(X1/X2) RNA expression itself is significantly increased after BAPN or LOX MO injections, suggesting that a decrease of LOX activity or protein synthesis both lead to an increase in its mRNA expression as a compensatory mechanism (Fig. S10).

The results show some differences in gene expression and phenotypes when comparing chemical inhibition of LOX and translation inhibition by MO. The two chemical inhibition drugs (BAPN and PXS) are irreversible and target the active LOX protein. These also hypothetically include all eight LOX proteins in the *S. purpuratus*, even though many of these do not seem to be expressed in early development. Differences in the effects of BAPN and PXS come from differences in their mechanism of action and specificity. We also see differences in gene expression of LOX-inhibited embryos when comparing chemical inhibitors and MOs. The MO target specific translation of LOXL(X1/X2) before it becomes an active protein. However, although MOs are injected into embryos minutes after fertilization, the maternal egg could still have active LOX protein already translated. In addition, it is important to note when using this MO, the other LOX genes could be overexpressed to compensate for the loss of LOXL(X1/X2). An Amplex Red experiment done on LOXL(X1/X2) MO embryos did not show a decreased fluorescence (not shown). This partial inhibition of LOX translation suggests that some enzymatic activity may still be present, prompting further investigation into how LOX inhibition affects broader cellular processes such as glycolysis.

Many glycolytic pathway metabolites are disturbed through LOX inhibition at the gastrula stage. In addition, some of the mRNAs coding for metabolic enzymes involved in glycolysis decreased after LOX inhibition, while the mRNA coding for a regulator of glycolysis is upregulated. LOX has not been shown to have a direct link to glycolysis; however, two regulators of LOX (TGF-B and hypoxia) have. Hypoxia, particularly, can promote glycolysis so cells can generate ATP without the presence of oxygen – a phenomenon known as the Warburg Effect (Liberti and Locasale, 2016). In this metabolic shift, cancer cells rely on glycolysis for energy production, allowing cells to rapidly produce ATP, supporting rapid cell proliferation. For cancer cells, the Warburg effect is particularly advantageous, as it enables them to thrive in the hypoxic regions of tumors where blood supply and oxygen are limited (Liberti and Locasale, 2016). Since hypoxia elevates LOX activity, this can be coupled with the Warburg effect. The increased LOX activity leads to a stiffer ECM, which supports cell survival, migration, and invasion. This remodeling also alters cell signaling pathways, such as integrin signaling (Liberti and Locasale, 2016).

### Conclusions

The ECM plays a crucial role in dynamically orchestrating cell development by providing structural support and biochemical cues that regulate germ cell migration and identity. LOX contributes to this regulation by crosslinking collagen, thereby influencing ECM stiffness and integrity (Fig. S11). Inhibition of LOX disrupts these ECM dynamics, potentially altering germ cell interactions and impeding proper developmental signaling. By chemically inhibiting LOX activity in embryonic development and analyzing phenotypes, molecular changes, and metabolomic studies, we can better understand the specific effects of ECM modulation in developmental biology. Understanding this intricate relationship offers valuable perspectives on how ECM remodeling influences germ cell identity and development.

## MATERIALS AND METHODS

### Embryo culture

Adults of the sea urchin *S. purpuratus* were housed in aquaria with artificial seawater at 16°C. Sea urchin gametes were acquired by 0.5 M KCl injection into the coelomic cavity of the adults. Eggs were collected in filtered sea water (FSW), and sperm was collected dry. Embryos were cultured in FSW at 16°C until the appropriate stage.

### Chemical inhibition of LOX through BAPN

Embryos of *S. purpuratus* were cultured in FSW in six-well plates (8 ml final volume) and dosed with BAPN at either 100 µM or 500 µM at different stages of development (from either fertilization to blastula, or blastula to gastrula, or gastrula to larva). Embryos were imaged on the Zeiss Axioplan 2 microscope to visualize morphology. Approximately 100 embryos were placed in RLT lysis buffer (Qiagen) for qPCR analysis, while the rest were collected and fixed in 4% paraformaldehyde for *in situ* RNA hybridizations.

A similar process was used to inhibit LOX through the small molecular inhibitor PXS-4787. Embryos of *S. purpuratus* were cultured in FSW and dosed with differing concentrations (100 µM or 500 µM) of PXS-4787, obtained from Syntara Ltd, using the same time windows as for BAPN (Chaudhari et al., 2023).

### Lysyl oxidase MO and microinjections

Fertilized eggs were injected with a lysyl oxidase [LOXL(X1/X2)]-specific MO (500 µM, Gene tools) with the sequence of (5′-AAAGAAGTCCTTGCACCAGTTTTCC-3′). This translation blocking MO sequence was designed by GeneTools for specificity. The sequence was blasted in the *S. purpuratus* genome to test for possible off-target sites, and none were found except for the LOXL(X1/X2) site. Control embryos were injected with an irrelevant MO designed against the sea star *P. miniata* FoxY3 (5′-TGCGATTAGAATCAAAACGGAGTGA-3′). MOs were co-injected with a green-fluorescent dye (FITC at a final stock concentration of 1 mM) as previously described (Song and Wessel, 2007). Injected embryos were collected at the blastula, gastrula, and larva stages. Embryos were fixed in 4% paraformaldehyde for fluorescence *in situ* hybridization or stored in RLT lysis buffer (Qiagen) for qPCR.

### *In situ* RNA hybridization

Whole-mount *in situ* RNA hybridization was performed using a digoxigenin-labeled RNA probe, as previously described (Arenas-Mena et al., 2000). Primers used to amplify the DNA templates are described in Table 1. The resulting templates were incubated for at least 3 h with SP6 RNA polymerase and digoxigenin-labeled NTPs (Roche) according to the manufacturer's instructions, then digested with DNase. Color RNA *in situ* experiments were performed as previously described (Minokawa et al., 2004), and the alkaline phosphatase reaction was carried out for up to 8 h. For fluorescent *in situ* RNA hybridization experiments, signals were detected with a peroxidase conjugated antibody and a TSA plus cyanine 3.5 (Akoya Biosciences SKU NEL763001KT). DAPI was used for nuclear staining. Negative controls were made by incubating embryos without any probe, or with an irrelevant probe (from a different species, for example, from the sea star *P. miniata*). All other protocol steps were performed identically. Fluorescent images were recorded from a Nikon spinning disk confocal microscope Ti2, with fluorescence laser settings adjusted to the exclusion of background (i.e. so that negative samples were tuned to no fluorescence, experimental samples then being imaged at the same settings). Brightfield images were obtained using the Zeiss Axioplan 2 fluorescent microscope.

**Table 1.** Primers for *in situ* RNA hybridization

No.	Gene	Model ID	F/R	Sequence (5′-3′)
1	*Vasa*	NM_001146193.1	F	GTCGAGGAAGGGGTCGCGGGTTCAGCCCCTTTG
			R	GATCATTTAGGTGACACTATAGGATGAGGGCCAGTGGTTCCTGGATCAC
2	*FoxY*	XM_003725961.3	F	CACACACTACTGGATGATCAACCCAAGC
			R	GATCATTTAGGTGACACTATAGGGTAGCGATAGAAACCTTTGGTGGTGTCG
3	*Nanos2*	NM_001079555.1	F	GGCGTCAACGTCCCCGGTATGTCCCGATC
			R	GATCATTTAGGTGACACTATAGGATGTTACCCGGTACCATGTTTCCCCC

This table provides the sequences of the primers used to make the probes for *in situ* RNA hybridization. Forward (F) and reverse (R) primers are listed along with their respective target genes and accession numbers. The SP6 polymerase binding sites were added at the 5′ of each reverse primer.

### Immunofluorescence

Embryos were prepared and fixed the same way as for the *in situ* hybridization methods. Embryos were labeled with Vasa antibody (rabbit polyclonal directed against the N terminal domain of *S. purpuratus* Vasa, dilution 1:200) as previously described (Voronina et al., 2008; Oulhen et al., 2019). Laminin antibody (rabbit polyclonal from Boster antibody, against a conserved region of *S. purpuratus* laminin-beta-1 sequence, dilution 1:400 (LOC582206)

[QFDLEAVFHFTHLIMTFKSFRPKAMVIERSSDFGHTWKPYRYFAYDCAGSFPEVS

RAPIRNISQVICESRYSAVEPSTGGEVIFRVLPPNIPIEDPYSREVQDMIKVTNIRINITELHFLGDHLFDDGDEVFNKYYYALYEMVVRGSCHCFNHASRCVPADGMEFKQQMVNGQCECIHNTEGKNCERCEPFFNDQPWRPAGLRGENNECKRCNCNDHATSCHFDEAVYRLTNGASGGVCDNCLHNTVGRNCEQCKPFFFMHPDRDIRDPNICVPCNCD]. All fluorescent images were recorded from the Nikon Spinning disk confocal Ti2 microscope using 40× oil-immersion lenses.

### qPCR analysis

100 embryos were collected at various time points, and RNA was extracted using the Qiagen RNeasy micro kit according to manufacturer's instructions. cDNA was produced using Maxima First Strand cDNA Synthesis (Thermo Fisher Scientific). qPCR primers were designed using the Primer3 program (Rozen and Skaletsky, 2000). Experiments were run in technical and biological triplicates, normalized to ubiquitin (Song and Wessel, 2007). Student's *t*-test (two-tailed, two-sample equal variance *t*-test) was used to determine *P*-value significance (*P*-value <0.05 is annotated as ‘*’. Primers are described in Table 2.

**Table 2.** Primers used in qPCR analysis

No.	Gene	Model ID	F/R	Primers (5′-3′)
1	*Vasa*	NM_001146193.1	F	TCAACTACGACCTCCCAAGC
	* *		R	TCTCGCAATGTTAGCATCCTT
2	*Nanos2*	NM_001079555.1	F	GCAAGAACAACGGAGAGAGC
	* *		R	CCGCATAATGGACAGGTGTA
3	*Endo16*	NM_214519.1	F	GTGCAAAGCTTCGAAAGGAC
	* *		R	CTCCCTCAACAATGGCGTAT
4	*FoxY*	XM_003725961.3	F	TGCACTGCACTGACTCTGC
	* *		R	CTTTCCATTCCGTGGTGAAG
5	*Wnt8*	NM_214667.1	F	TGTCGTTCATTCAAGCCATC
	* *		R	TATCACTCGCCATTCGTTCA
6	*Nodal*	NM_001098449.1	F	GACAACCCAAGCAACCACG
	* *		R	CGCACTCCTGTACGATCATG
7	*Sm50*	NW_022145602.1	F	TAGCCTTTGCTACGGGTCAA
	* *		R	CTGAGGCGACGAAACTGAA
8	*Seawi*	NM_214600.1	F	GTGATGGTGTTGGTGACAGC
	* *		R	TATTGATGCGCTTCTTGACG
9	*Alx1*	XM_011663478.2	F	GTTGTTGGTCGAGTCCTGCT
	* *		R	TCAAACCGGCCCTAGACTC
10	*FoxA*	NM_001079542.1	F	GCTGCGATGTCGATGAGATA
	* *		R	CACCGTTGTTGATTTTGACG
11	*Laminin-beta-1*	XM_030972670.1	F	GGCAGCTCATCCTTGCTGGAAGCG
	* *		R	CACCCAACATCCCCATCGA
12	*Pitx2*	XM_777251.4	F	ACATTTCACCAGCCAGCAAC
	* *		R	TCAAGTTACACCACGCACAGA
13	*FoxC*	XM_003727734.3	F	GAAAATCACCCTCAACGGAAT
	* *		R	CCCCTTGCCTGGTTTCTTAT
14	*FoxY*	XM_030975701.1	F	TTGAGTGAGAGGTTGTGACGA
	* *		R	ATCGTAATGGCGATCCAGAG
15	*Eya*	XM_030982769.1	F	TACCAGTTAGCGCCGTTACC
	* *		R	TTGACCTTGACGCCCTCTTC
16	*SoxE*	XM_781716.5	F	TCCTCCTCAATACCCCT
	* *		R	GAGTGTTGCTGTGCAGGGTA
17	*Six1/2*	NM_001281755.1	F	GTCTGCGAGGTTCTCCAACA
	* *		R	CGCCTTGGCTTTGAGAACAC
18	*Dach*	XM_030981289.1	F	GAGACTGAGCATCACTCCGG
	* *		R	TGGTGAGCAGTTTACAGCGG
19	*Pax6*	XM_795141.5	F	CGGACTCTACCCGACAGAAG
	* *		R	GGTCGTATGCTCCCTGTCTC
20	*ADAMTSL-5*	XM_011678898.2	F	AATGCAAACGGGTGAAAGGC
	* *		R	TGTTCGAAGCTTAGGGCAGG
21	*Bamacan*	XM_030985548.1	F	TCGAGAGTTGAAAGGTCGCT
	* *		R	AATGGTACCCTTGGCATCCA
22	*Col4a1*	NM_214466.1	F	TTTGTGCCAGGGAGATTTGC
	* *		R	AAACTGGCTAGGCTCCTCTG
23	*Dermatan*	XM_782879.5	F	TCTGCAGTTCCTTGAGAGCA
	* *		R	TAACGGGTGAGCTGTCCAAT
24	*Integrin-A6*	XM_030990668.1	F	GTGCTACTTCATGTCTGCCG
	* *		R	TCGGTGTACTTGAGCCTTGT
25	*MMP-24*	XM_786523.5	F	ACTCACCTGGGCAATCAAGA
	* *		R	GCCATCGAAAGCATAACCGT
26	*Reelin*	XM_031000304.1	F	CAGCCTTCAAGTGGAGGAGA
	* *		R	TATTGCATGCCTCGCCAATC
27	*Dystroglycan*	XM_781496.5	F	CCTTAGCTGCAGGCAGGGG
	* *		R	CGCGCCGTATGCCGGTTATAGTCAG
28	*GAPDH*	XM_030986815.1	F	GTGGTGGCAGTCAATGATCC
	* *		R	GACCACGTACTCAGCTCCAT
29	*Mannose Isomerase*	XM_775211.5	F	CAAAGTGCTGTCCGTGAACA
	* *		R	AGACAGGGAAGCAGCATTCT
30	*Pyruvate Kinase*	XM_031000415.1	F	CGCAACTCTGGAATCGTCTG
	* *		R	CCTTCGTGTCCAGAGCTACA
31	*Glutaredoxin*	XM_782286.5	F	GCTAGCGCTGGTCTTAAGGA
			R	AATTTCCCGCTTTCCTGCAA
32	*Ubiquitin*	XM_030976721.1	F	CACAGGCAAGACCATCACAC
			R	GAGAGAGTGCGACCATCCTC

This table provides the sequences of primers used for quantitative PCR (qPCR) analysis. Forward (F) and reverse (R) primers are listed along with their respective target genes and accession numbers. Primer sequences were designed for specificity and efficiency in amplifying the target regions. Primer validation was performed through melt curve analysis.

### LOX gene identification

Sequences of LOX orthologs were identified through gene searches in Echinobase (Telmer et al., 2024). Open reading frames were found through Expasy (Gasteiger et al., 2003). Protein domains were identified with InterProScan (Jones et al., 2014).

### PCR on 5′UTR sequence

cDNA was made from *S. purpuratus* RNA isolated from various stages of embryonic development, using the Maxima first strand cDNA kit. DNA was then amplified with the GoTaq Master Mix (Fisher Scientific). Three primers were designed in the beginning, middle and end of the 5′UTR with two reverse primers located after the start codon. The following primers were used: F1, 5′-AGCTTTGATCGTAAGCCTACTTGAG-3′; F2, 5′-GTAAAGTAGTTTCGGTTTAAACCTCAAC-3′; F3, 5′-GCACGAGTTGACGACTACTTCAAAATG-3′; R1, 5′-CCGCACAAGCTCATACCACAATAGACGAATGC-3′; R2, 5′-GAAACCAGGAACCATGCACTTTACCC-3′.

### LOX activity assays

Amplex Red reagent (Thermo Fisher Scientific) (Fernando Rodriguez-Pascual, 2022) was used to measure the H_2_O_2_ released from cells at different embryonic stages. Control and LOX-inhibited embryos (by means of PXS-4787 and BAPN inhibition at 500 µM) were collected at blastula, gastrula, and larva stage. A working solution of 100 µl per well was created with 10 mM Amplex Red reagent and 10 U/L horseradish peroxidase all dissolved in Krebs-Ringer bicarbonate buffer. 20 µl of embryos (approximately 200 embryos) were added to each well, and 100 µl of reaction mix was added to begin the reaction. Microplate readings on Molecular Device's SpectraMax iD3 were taken at 2 min, 15 min, 30 min, and 60 min. Base levels of H_2_O_2_ in the embryos was accounted by subtracting values from the control wells with no reagents added.

### Allysine-binding chemical probe

A chemical probe binding to allysine was provided by the lab of Helma Wennemers, Laboratory of Organic Chemistry, D-CHAB, ETH Zurich (Hiebert et al., 2024). The probe is based on a previously reported probe to monitor LOX-mediated tissue remodeling and consists of a standing fluorophore attached to the collagen-reactive peptide Ahx-[POG]_3_-AopPG-[POG]_3_ [P=proline, O=(4*R*)-hydroxyproline, G=glycine, Aop=(4*S*)-aminooxyproline] (Hiebert et al., 2024; Aronoff et al., 2021). Reagents were diluted to a stock concentration of 1 mM, and dilutions were made in either nuclease free water or filtered sea water (for embryos that were methanol fixed or alive, respectively) to a concentration of 100 µM. Around 20 embryos were placed in each well of a 96-well plate, and 100 µl of total reagent was added. Live embryos were soaked for 4 h in the allysine-binding probe, washed in filtered sea water, then imaged immediately with the Nikon Spinning disk confocal Ti2 microscope. Methanol-fixed embryos were soaked for 4 h in the Bioprobe and PBST, washed in PBST, then imaged immediately with the Nikon Spinning disk confocal Ti2 microscope.

### Metabolomics

Metabolomic profiling was conducted using Novogene's untargeted metabolomics platform. 100 µl of pelleted embryos treated with BAPN or PXS, as well as two technical replicates of controls, were collected at the gastrula stage. Embryos were placed in an extract solvent consisting of methyl tert-butyl ether/methanol 10:3 v:v, vortexed and spun, and the supernatant was collected and sent to Novogene. The samples were processed by UHPLC-MS/MS analysis on a high-resolution mass spectrometer. Metabolites were identified and quantified using a combination of KEGG, Human Metabolome Database, and public spectral databases. Data processing and statistical analysis were performed with multivariate and univariate approaches to identify significant metabolic changes.

## Supplementary Material

10.1242/biolopen.062198_sup1 Supplementary information
