# Supplementary material for: Collagen processing is essential for germ cell identity
Source: Biol Open. 2025 Nov 20;14(11):bio062198. doi: 10.1242/bio.062198 (PMC12673967; doi:10.1242/bio.062198)
Supplement: Supplementary information [file biolopen-14-062198-s1.pdf]

A

| **Echinobase Name *S. purpuratus*** | **LOC #** | **NCBI #** | **New Name** |
|---|---|---|---|
| Lysyl oxidase-like 2 (LOXL2) | LOC110979849 | NM_001079547.1 | LOXL-2A |
| Uncharacterized LOX (X1) | LOC100891205 | XP_003730138.1 | LOXL-X1 |
| Uncharacterized LOX (X2) | LOC100891205 | XM_030982711.1 | LOXL-X2 |
| Lysyl oxidase homolog 2 | LOC100893720 | XM_030975789.1 | LOXL-2B |
| Lysyl oxidase homolog 2 | LOC100889475 | XM_030976908.1 | LOXL-2C |
| Lysyl oxidase homolog 3A-like | LOC105438519 | XM_030989818.1 | LOXL-3 |
| Lysyl oxidase homolog 2 | LOC579947 | XM_011682351.2 | LOXL-2D |
| Lysyl oxidase homolog 2 | LOC100891297 | XM_003729241.3 | LOXL-2E |
| Lysyl oxidase homolog 2 | LOC752624 | XM_001179710.4 | LOXL-2F |

**Fig. S1. Annotating LOX proteins in *S. purpuratus***

Biology Open • Supplementary information

```
X2          MGPFNTGHSSHFGQLLKGPHVQLNDFVGLFFLIIGLQCYAAQAHT---------------
X1          MGPFNTGHSSHFGQLLKGPHVQLNDFVGLFFLIIGLQCYAAQETSRLRLISGATPNEGRV
            ******************************************  :

X2          ------------------------------------------------------------
X1          EVDVGDGRGWGTICDNGWGYNDASVVCKQIGYPAATFSTPGARFGGNPTLPILLENVACS


X2          ----TIDECPSANPSASCDHTNDAGVKCMVPGFLGCFSLLTIGSRAWTIPENSNDACKAQ
X1          PSHTTIDECPSANPSASCDHTNDAGVKCMVPGFLGCFSLLTIGSRAWTIPENSNDACKAQ
                ********************************************************

X2          CKDLDYRYAGMSGTSCRCGNNRLFYFYNQYPDYYCNSNCKGDATQLCGNTVSSYFSVFDT
X1          CKDLDYRYAGMSGTSCRCGNNRLFYFYNQYPDYYCNSNCKGDATQLCGNTVSSYFSVFDT
            ************************************************************
```

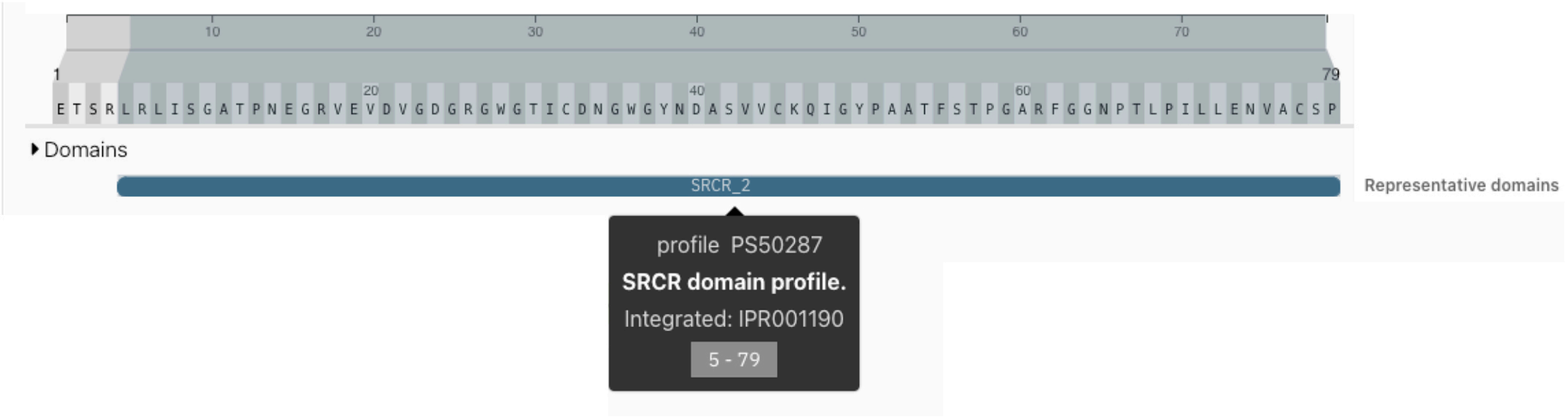


**Fig. S2. Alignment of LOXL-X1 and LOXL-X2 isoform**

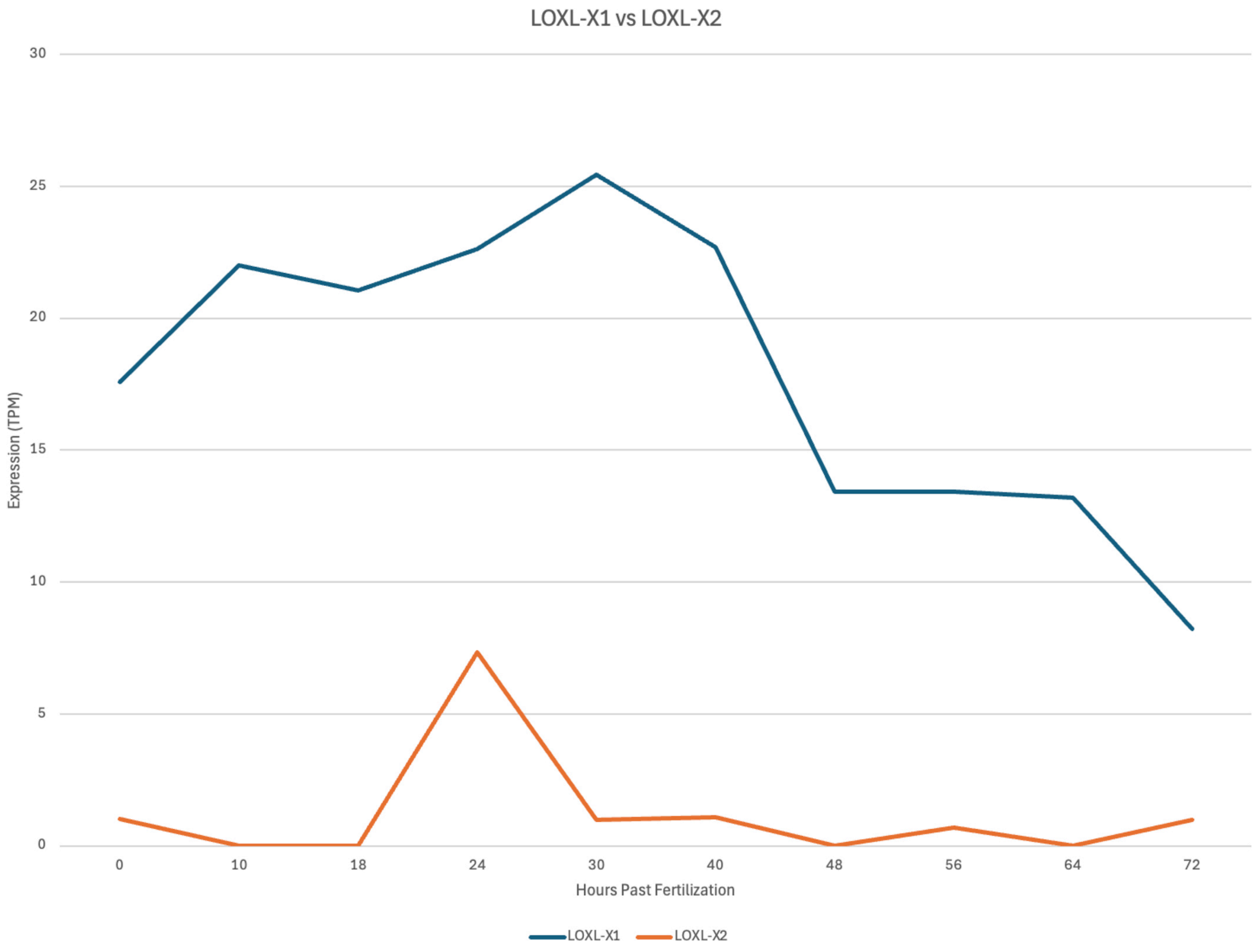


**Fig. S3. mRNA Expression of LOXL-X1 and LOXL-X2 isoform**

Biology Open • Supplementary information

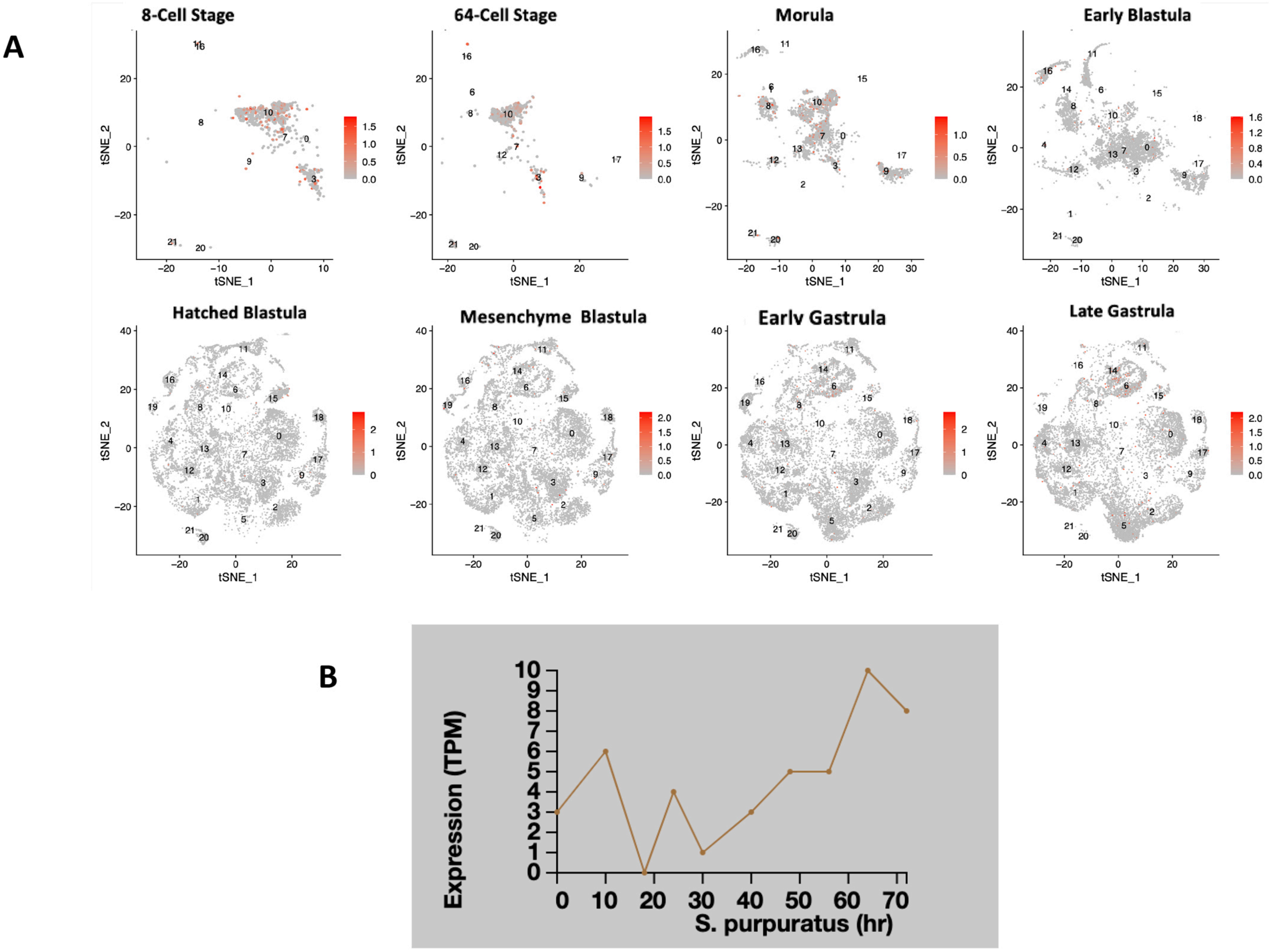


**Fig. S4. mRNA expression of LOXL-2B**

Biology Open • Supplementary information

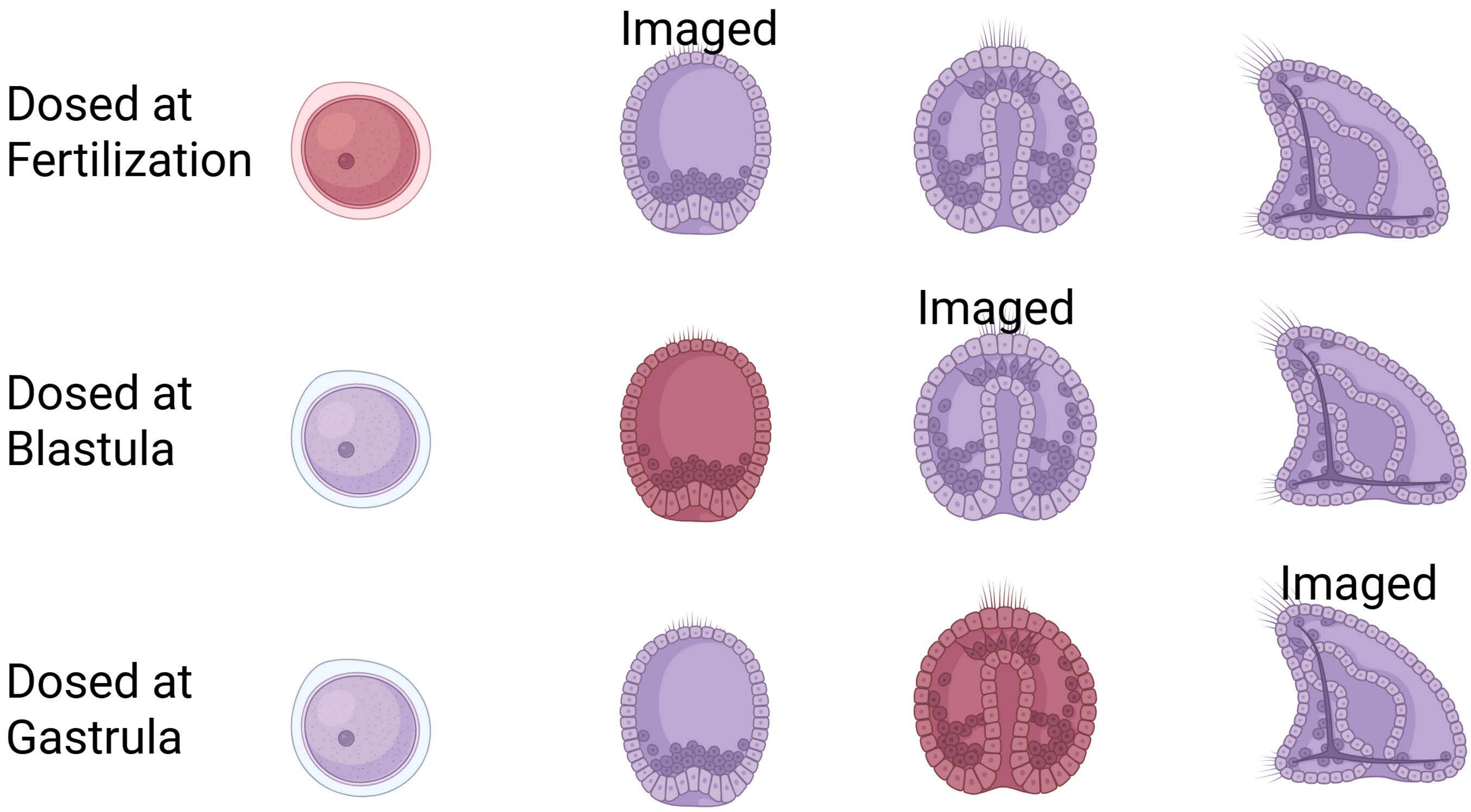


**Fig. S5. Dosage scheme for all experiments involving soluble reagents (BAPN and PXS-4787).** Created in BioRender by Oulhen, N. (2025). https://BioRender.com/d1fvshd. This figure was sublicensed under CC-BY 4.0 terms.

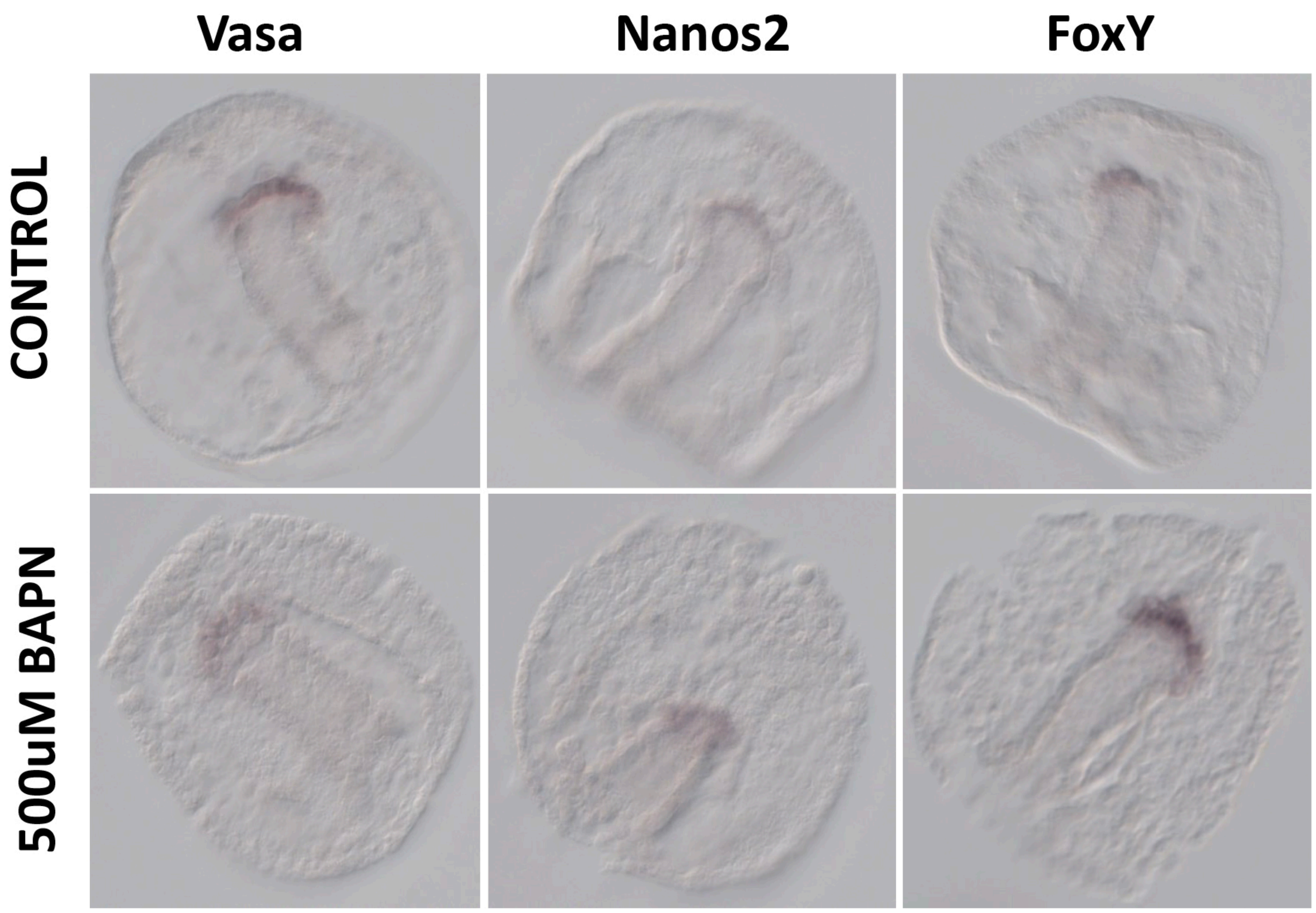


**Fig. S6. In situ RNA hybridization after BAPN treatment**

Biology Open • Supplementary information

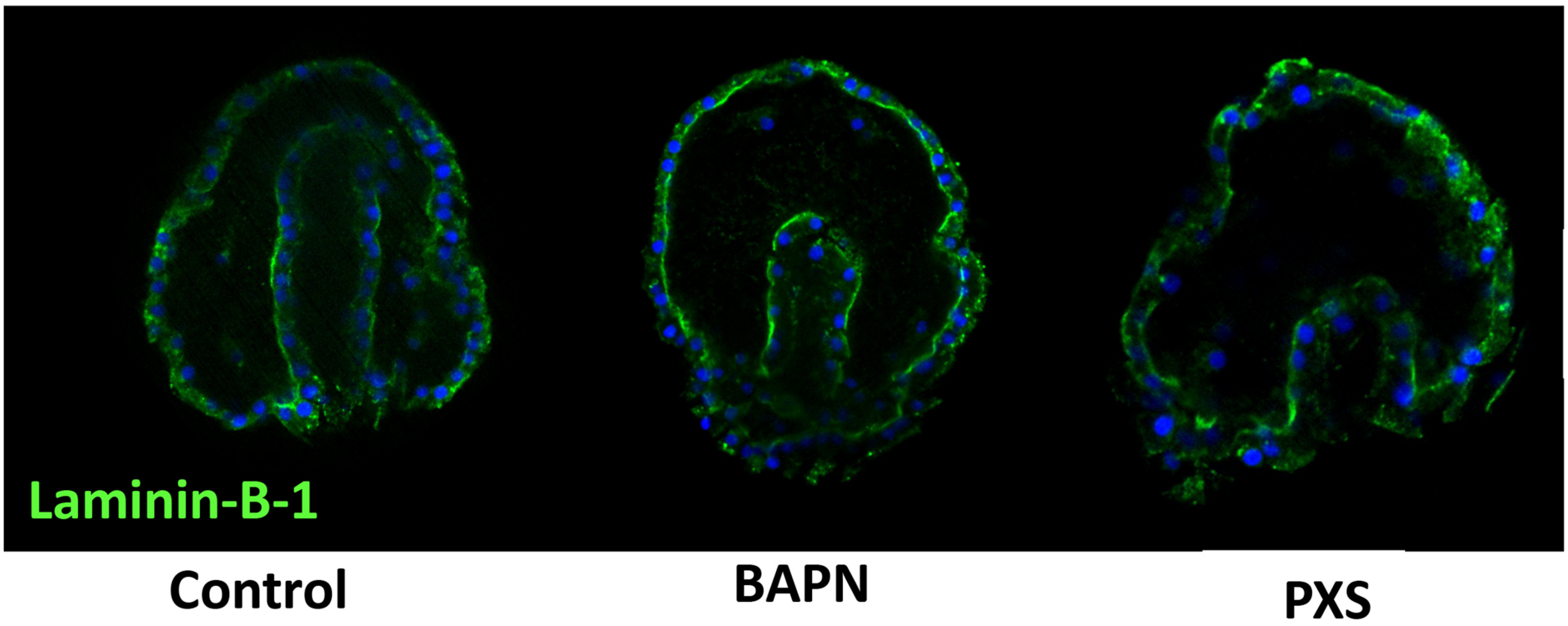


**Fig. S7. Immunofluorescence of Laminin-beta-1 on LOX inhibited embryos**

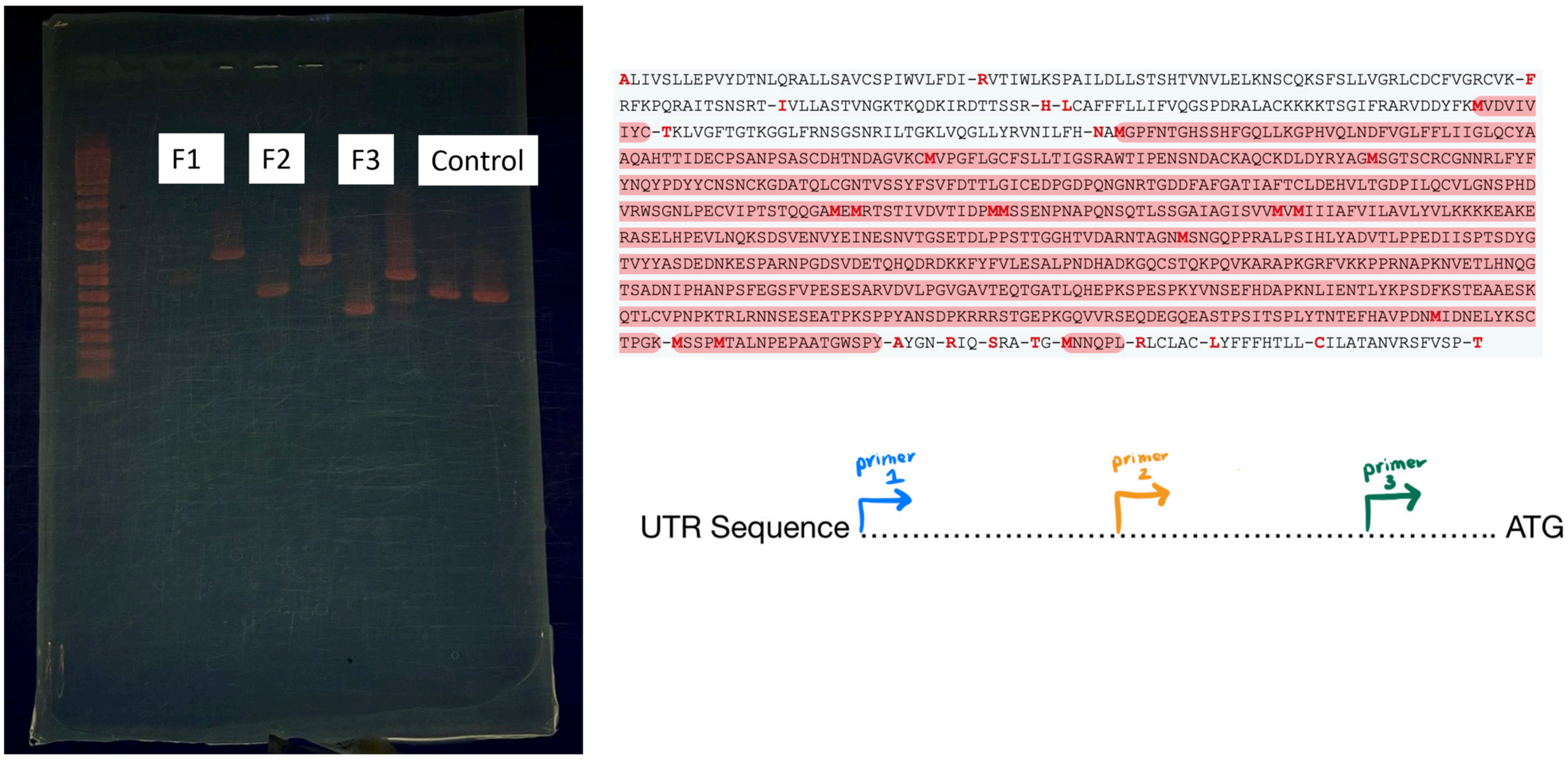


**Fig. S8. Characterization of LOXL(X1/X2) 5’UTR**

Biology Open • Supplementary information

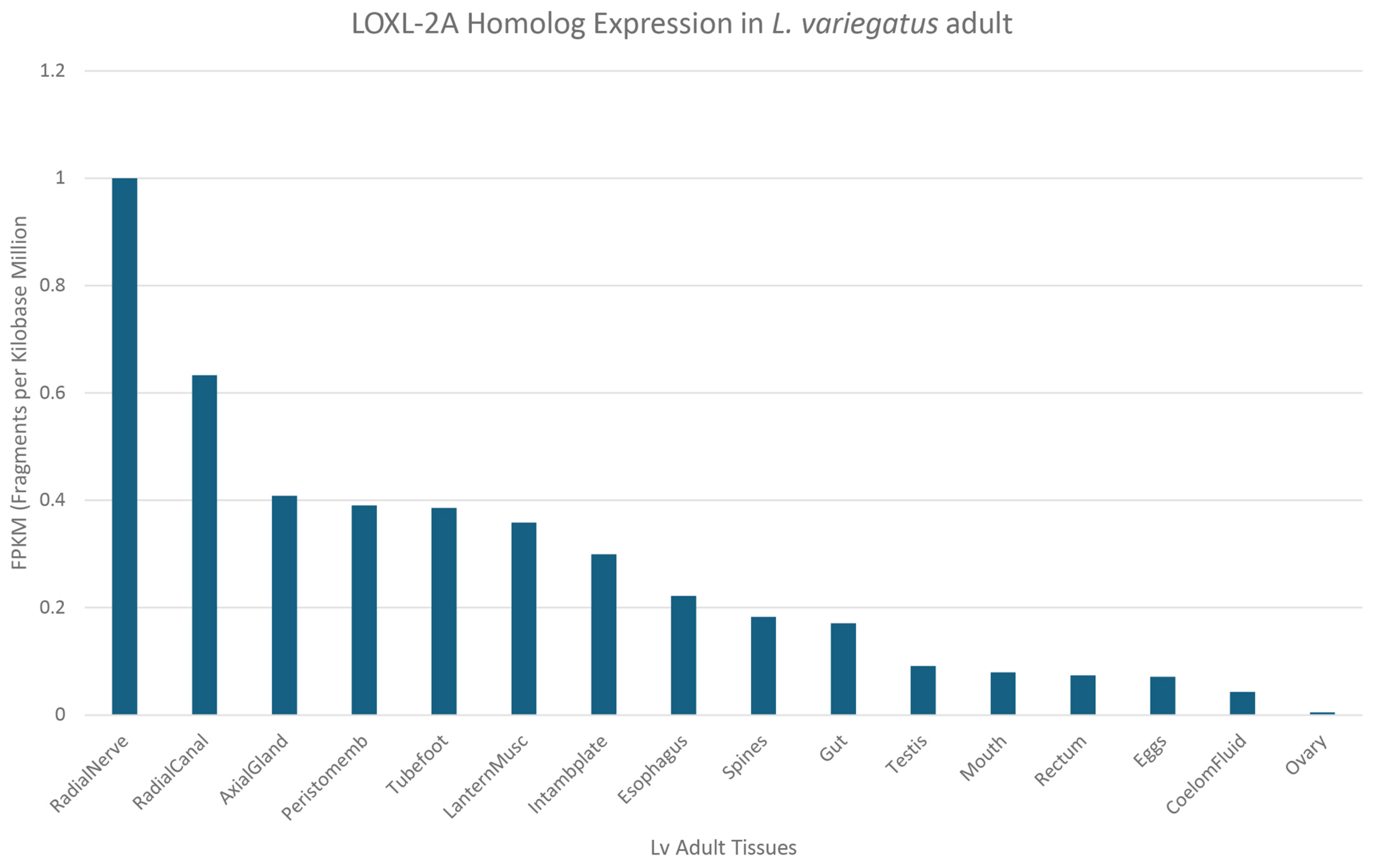


**Fig. S9. LOXL-2A expression in body structures of the adult *Lv* sea urchin.**

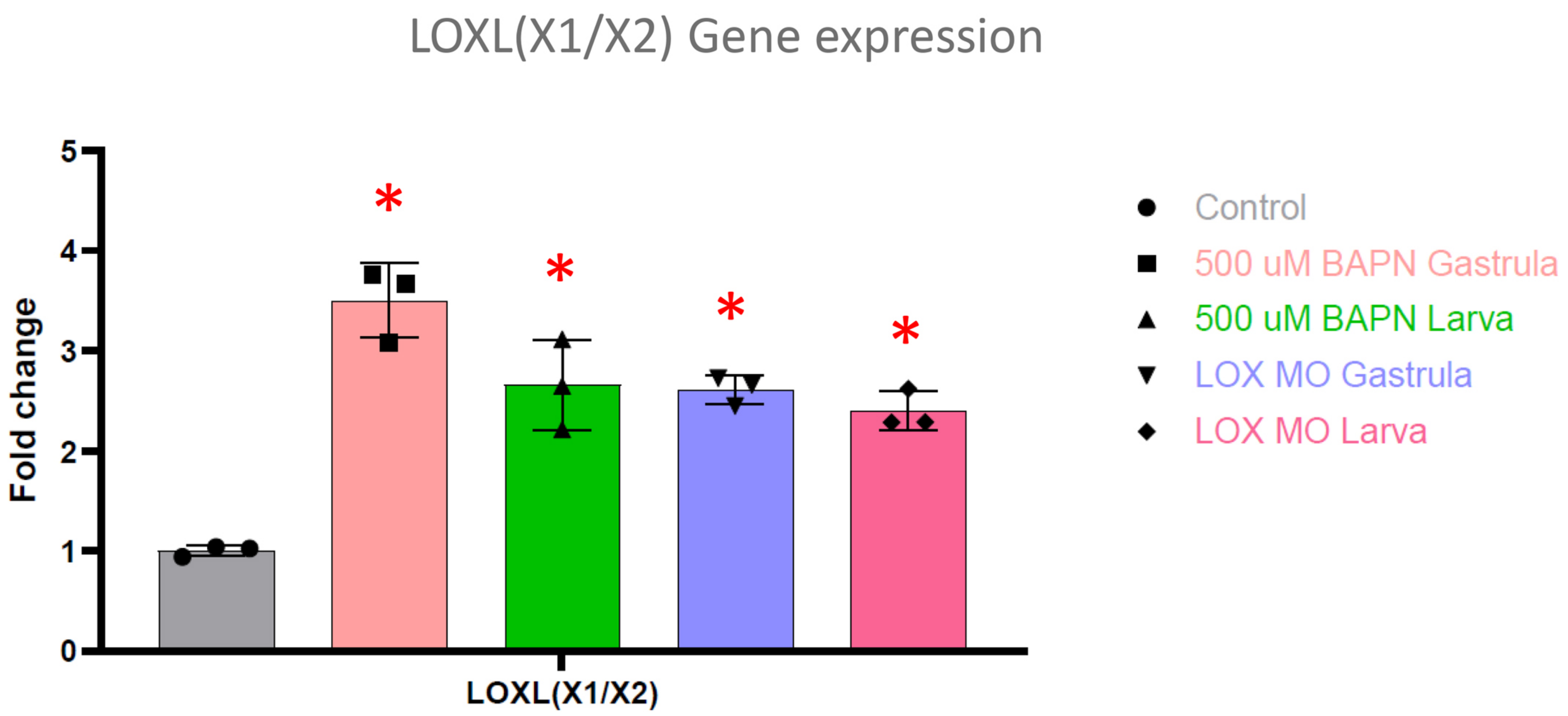


**Fig. S10.** **LOXL(X1/X2) RNA expression increased after BAPN treatment and LOX MO injection.**

Biology Open • Supplementary information

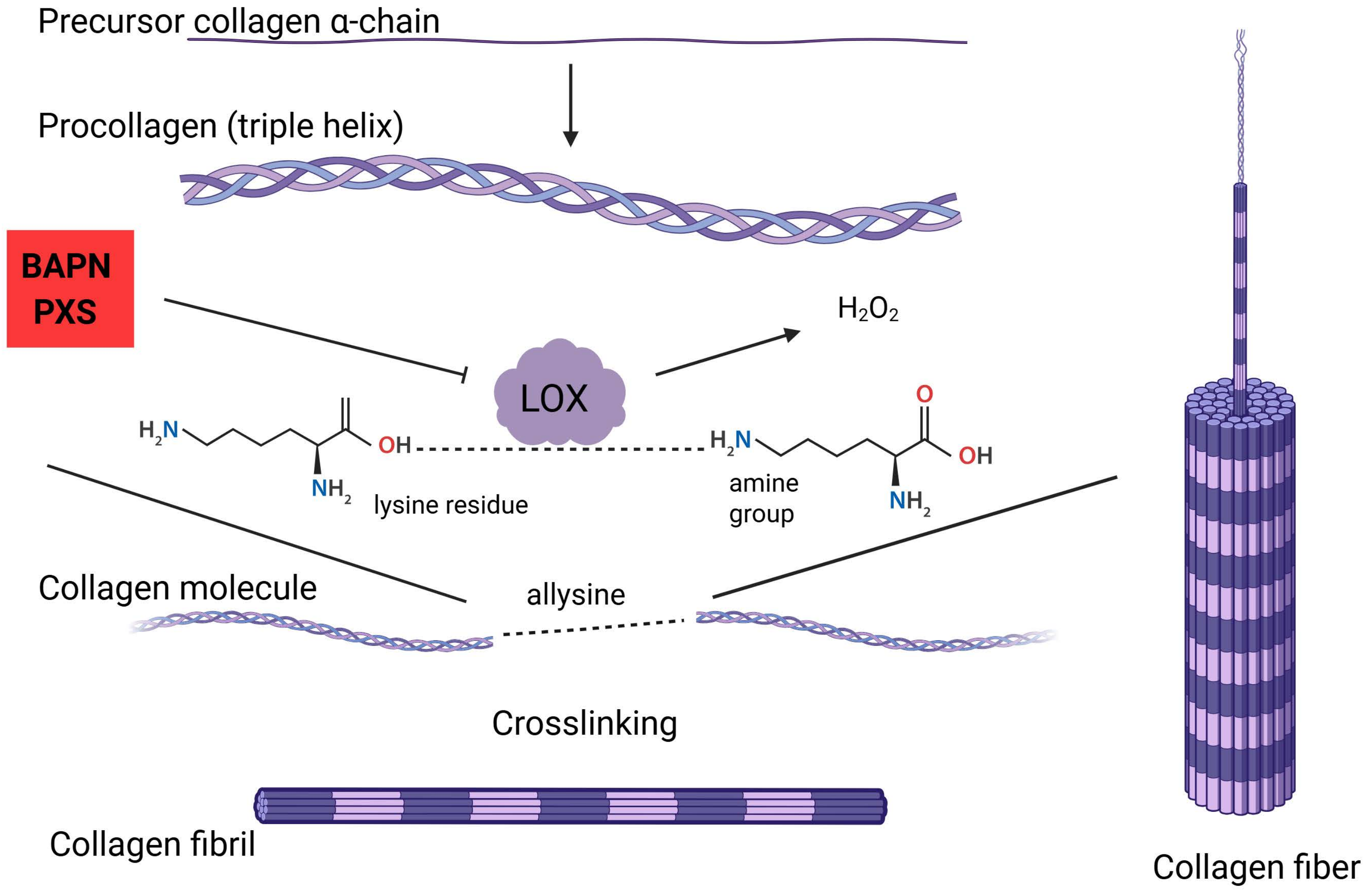


**Fig. S11. Collagen processing and stability is central to a stable ECM.** Shown here are key determinants to stability experimentally tested in this study. Lox activity - a central processing step. BAPN and PXS - inhibitors of Lox. $H_2O_2$ as a metric for Lox activity. The purple color is indicative of the development of collagen fibers from a single monomer to a complex, crosslinked multimer. Created in BioRender by Oulhen, N. (2025). https://BioRender.com/a96xmlc. This figure was sublicensed under CC-BY 4.0 terms.

Biology Open • Supplementary information
